# Structural analysis reveals TLR7 dynamics underlying antagonism

**DOI:** 10.1038/s41467-020-19025-z

**Published:** 2020-10-15

**Authors:** Shingo Tojo, Zhikuan Zhang, Hiroyuki Matsui, Masahiro Tahara, Mitsunori Ikeguchi, Mami Kochi, Mami Kamada, Hideki Shigematsu, Akihisa Tsutsumi, Naruhiko Adachi, Takuma Shibata, Masaki Yamamoto, Masahide Kikkawa, Toshiya Senda, Yoshiaki Isobe, Umeharu Ohto, Toshiyuki Shimizu

**Affiliations:** 1 Sumitomo Dainippon Pharma Co., Ltd., 3-1-98 Kasugade-naka, Konohana-ku, Osaka, 554-0022 Japan; 2 Graduate School of Pharmaceutical Sciences, The University of Tokyo, 7-3-1 Hongo, Bunkyo-ku, Tokyo, 113-0033 Japan; 3 Graduate School of Medical Life Science, Yokohama City University, 1-7-29, Suehiro-cho, Tsurumi-ku, Yokohama, Kanagawa 230-0045 Japan; 4 RIKEN SPring-8 Center, 1-1-1 Kouto, Sayo Hyogo, 679-5148 Japan; 5 Department of Cell Biology and Anatomy, Graduate School of Medicine, the University of Tokyo, 7-3-1 Hongo, Bunkyo-ku, Tokyo, 113-0033 Japan; 6 Structural Biology Research Center, Institute of Materials Structure Science, High Energy Accelerator Research Organization (KEK), 1-1 Oho, Tsukuba, Ibaraki 305-0801 Japan; 7 Division of Innate Immunity, Department of Microbiology and Immunology, The Institute of Medical Science, The University of Tokyo, 4-6-1 Shirokanedai, Minato-ku, Tokyo, 108-8639 Japan

**Keywords:** Toll-like receptors, Electron microscopy, X-ray crystallography

## Abstract

Toll-like receptor 7 (TLR7) recognizes both microbial and endogenous RNAs and nucleosides. Aberrant activation of TLR7 has been implicated in several autoimmune diseases including systemic lupus erythematosus (SLE). Here, by modifying potent TLR7 agonists, we develop a series of TLR7-specific antagonists as promising therapeutic agents for SLE. These compounds protect mice against lethal autoimmunity. Combining crystallography and cryo-electron microscopy, we identify the open conformation of the receptor and reveal the structural equilibrium between open and closed conformations that underlies TLR7 antagonism, as well as the detailed mechanism by which TLR7-specific antagonists bind to their binding pocket in TLR7. Our work provides small-molecule TLR7-specific antagonists and suggests the TLR7-targeting strategy for treating autoimmune diseases.

## Abstract (web summary)

A series of Toll-like receptor 7 (TLR7)-specific antagonists and extensive structural analysis reveal the open conformation of the receptor and the structural basis of TLR7 antagonism. One of the compounds shows efficacy in treating mouse model of systemic lupus erythematosus.

## Introduction

TLR7 recognizes pathogenic single-stranded RNAs (ssRNAs) and plays a crucial role in the innate immune response to viral infections, such as human immunodeficiency virus 1 and influenza virus^1,2^. Our previous structural studies of TLR7 elucidated the homo-dimeric activated form of TLR7^3^. The activated TLR7 dimer contains two distinct ligand-binding sites. Small-molecule agonists, including imiquimod, guanosine and 2’,3’-cGMP bind to site 1 in the dimerization interface and directly induce the formation of the activated TLR7 dimer, while uridine-containing ssRNAs bind to site 2 and allosterically enhance the binding affinity of site 1 ligands for TLR7^3,4^.

TLR7 is strictly localized to the endosomal compartment to avoid undesirable endogenous RNA-mediated activation^5,6^. In fact, excessive activation of TLR7 is considered to be involved in the pathogenesis of several autoimmune diseases such as systemic lupus erythematosus (SLE)^7–10^. In contrast, the participation in SLE of other members of the TLR7 family, TLR8 and TLR9, remains unclear or controversial^11,12^. These findings suggested that selective targeting of TLR7 would be useful for the treatment of SLE. Several TLR7 inhibitors, including oligonucleotides and antimalarial agents, have been reported^13,14^. Antimalarial agents, represented by hydroxychloroquine (HCQ), inhibit TLR7 and also TLR9 via indirect mechanisms that do not involve direct binding to TLRs^14^. To date, potent and selective TLR7 inhibitors have not been discovered.

We present here the development of potent TLR7-specific antagonists via the modification of TLR7 agonist. Using cryo-electron microscopy (cryo-EM) combined with X-ray crystallography, we reveal that the structural equilibrium between open and closed conformations that underlies TLR7 antagonism. In addition, high-resolution cryo-EM structure enables us unambiguously to visualize an antagonist binding sites inside the open-form structure of TLR7 and the recognition mechanism of TLR7 antagonists at the atomic level. Finally, we examine the therapeutic efficacy of Cpd-7 in lupus-prone NZB/W F1 mice, which shows that oral administration of this promising compound can protect mice against deadly autoimmunity.

## Results

### Design of TLR7 antagonists based on its agonist

**Fig. 1:**
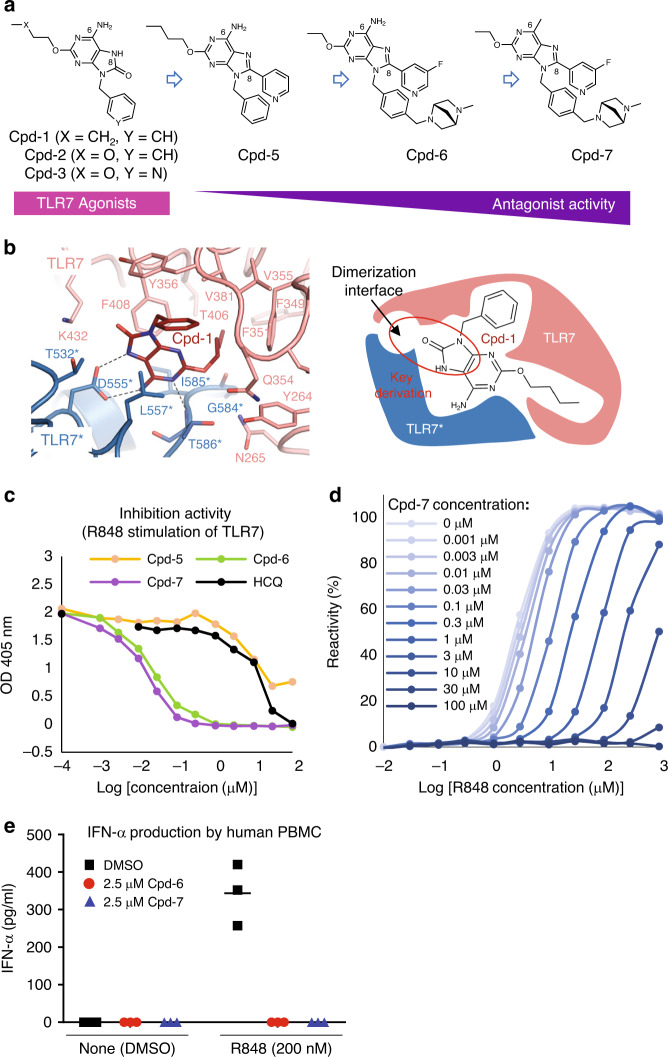
Design strategy of TLR7 antagonists. **a** Flow-chart of the conversion from TLR7 agonists to antagonists. The chemical structures of these compounds are shown. **b** The binding mode of Cpd-1 to TLR7 site 1 in its activated form is shown on the left. TLR7 is shown in ribbon-and-stick representations. Two TLR7 protomers in the dimeric structure are painted salmon and aquamarine, respectively. Dashed lines indicate hydrogen bonds. On the right is a schematic view of Cpd-1 binding to site 1. The space inside the dimerization interface and the key derivation part of Cpd-1 are indicated by a red ellipse. **c** Dose–response curves for the inhibition of human TLR7 activity by Cpd-5, Cpd-6, Cpd-7 and HCQ (positive control) measured by reporter gene assay with R848 (200 nM) as stimulating agent. Data are representative tests with three replicates. **d** Dose–response relationship between Cpd-7 and R848 measured by human TLR7 reporter gene assay. TLR7-expressing cells were incubated with Cpd-7 (0–100 μM), together with 0–1000 μM of R848. Data are representative tests with three replicates. **e** TLR7 inhibition assay in primary human PBMCs. PBMCs were pretreated by DMSO, Cpd-6 or Cpd-7 for 3 h before 20 h stimulation by DMSO (none) or R848. Secreted IFN-α levels quantified by ELISA were shown. Dot plots of the data with mean (*n* = 3 per group) are shown.

To identify TLR7-specific antagonists, we assumed that a TLR7-specific agonist could serve as a scaffold for development of TLR7-specific antagonists. We have previously reported a series of 8-oxoadenine derivatives (8-OAs) represented by Cpd-1, Cpd-2, and Cpd-3 as specific TLR7 agonists^15–18^. Therefore, we used these compounds as a starting point for further development (Fig. 1a). We confirmed the binding mode of 8-OAs by crystallography (Supplementary Table 1). These compounds bound to TLR7 site 1 (Fig. 1b) and the overall structures of the complex were similar to those of known TLR7/agonist complexes^4^ (Supplementary Fig. 1a–c). The structure-activity relationships (SAR) of the 8-OAs indicated that both 6-amino and 8-oxo groups were important in TLR7 agonism^19^ (Fig. 1a), but the 8-oxo group was too far to interact with TLR7 protein, resulting in a space in which other substituents might be introduced (Fig. 1b). We hypothesized that the substitution of the 8-oxo group in 8-OAs would convert an agonist into an antagonist. We synthesized Cpd-5 with an 8-pyridyl group, showing low solubility (0.0003 mg/ml at pH 7.4 buffer) and modest TLR7 inhibitory potency (IC_50_ = 5.0 μM) comparable to HCQ without causing any inhibition of either TLR8 or TLR9 and activation of TLR7 (Figs. 1a, 1c and Supplementary Fig. 2). We further optimized the C2 side chain and 8-pyridyl moiety of Cpd-5, and also introduced an amine unit into the para-position of the 9-phenyl group in order to enhance solubility and potency with reference to our SAR of 8-OAs^18^ (Fig. 1a). After these optimizations, Cpd-6 displayed potent TLR7 inhibitory activity (IC_50_ = 25 nM) with a high selectivity for TLR7 over TLR8 and TLR9 (Fig. 1c and Supplementary Fig. 2). We also synthesized a 6-methyl derivative, Cpd-7, based on the structure of Cpd-6. This modification increased the inhibitory activity (Fig. 1d and Supplementary Fig. 2; IC_50_ = 15 nM). Both Cpd-6 and Cpd-7 showed good solubility (>0.15 mg/ml at pH 7.4 buffer). The dose-response relationship between the inhibitors and R848 showed that both Cpd-6 and Cpd-7 acted as antagonists of TLR7 (Fig. 1d and Supplementary Fig. 3).

We also conducted the inhibition assays in primary human peripheral blood mononuclear cells (PBMCs) in which plasmacytoid DCs, but not other immune cells, produce interferon-α (IFN-α) in TLR7 dependent manner^20^. The result showed that both Cpd-6 and Cpd-7 completely blocked R848-induced interferon-α (IFN-α) production at 2.5-μM concentration (Fig. 1e), while neither Cpd-6 nor Cpd-7 blocked the TLR2-dependent TNF-α production from human PBMCs at the same concentration (Supplementary Fig. 4), suggesting that these inhibitors are not cytotoxic and specifically inhibit TLR7 response in human primary immune cells. These results demonstrate that TLR7 antagonist activity of Cpd-6 and Cpd-7 can be confirmed both in NF-κB/SEAP reporter gene assay and the assay using PBMC.

### Structural analysis showed TLR7 dynamics upon antagonist binding

**Fig. 2:**
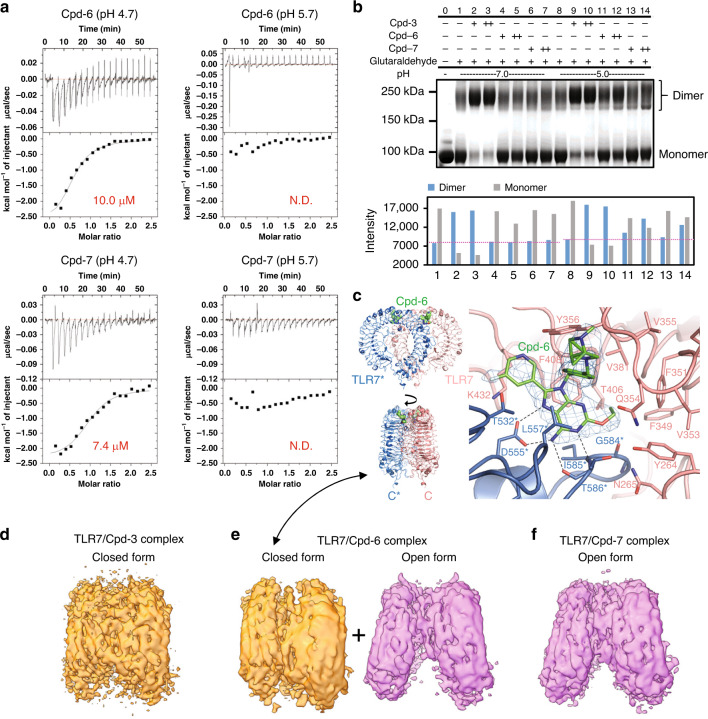
Cryo-EM analyses reveal TLR7 conformational dynamics induced by antagonists. **a** ITC titrations of Cpd-6 or Cpd-7 to TLR7 recombinant proteins at pH 4.7 or 5.7. Dissociation constant values (*K*_D_) are shown in red. N.D. refers to ‘not detected’. **b** Crosslinking assay of ligand-induced dimerization of TLR7 at neutral or acidic pH conditions (upper panel). Quantitative analysis of the SDS-PAGE gel image (lower panel). The densities of the band areas corresponding to the dimeric and monomeric TLR7 were calculated respectively. The experiment was replicated twice. **c** Crystal structure of TLR7/Cpd-6 complex. Overall structure in cartoon (left) and detailed view of Cpd-6 recognition at site 1 (right). The 2Fo-Fc map of Cpd-6 countered at 1 σ is shown as a blue mesh. Dashed lines indicate hydrogen bonds. **d**–**f** Cryo-EM density maps of the TLR7/Cpd-3, TLR7/Cpd-6 and TLR7/Cpd-7 complexes obtained by 200 kV microscopes. The closed and open forms of TLR7 dimer are shown as orange and magenta, respectively. The closed form of TLR7 dimer is similar to that observed in the crystal structure of the TLR7/Cpd-6 complex shown in **c**.

To understand the mechanism of action of our developed TLR7-specific antagonists, we conducted biochemical and structural studies using the extracellular domain of TLR7 from monkey. ITC analysis confirmed the direct binding of Cpd-6 and Cpd-7 to TLR7 and that the binding depends on an acidic pH (Fig. 2a). The critical pH near 5.0 coincides well with the pH inside mature endo-lysosomes where TLR7 resides. We next conducted a crosslinking assay to monitor ligand-induced TLR7 dimerization and performed its quantitative analysis (Fig. 2b). The potent agonist Cpd-3 induced almost complete dimerization under both neutral and acidic conditions. TLR7 in the absence of ligands remained mostly monomeric, but the extent of dimerization in the presence of Cpd-6 and Cpd-7 increased only in acidic conditions, demonstrating that direct binding of Cpd-6 and Cpd-7 to TLR7 induced dimerization in an acidic condition.

To our surprise, crystallographic study of the TLR7/Cpd-6 complex revealed that this complex adopted an activated dimeric structure and Cpd-6 bound to site 1 with a recognition mode very similar to that of Cpd-1 (Fig. 2c, Supplementary Fig. 1d and Supplementary Table 1). The C8 substitution group in Cpd-6 showed weak electron densities, indicating the high flexibility of this part (Fig. 2c). The static crystal structure showed one aspect of the binding mode, but its inhibitory mechanism remained elusive. We thus decided to take advantage of electron microscopy to capture the possible conformational dynamics associated with TLR7 antagonism. Crosslinked dimeric TLR7 proteins prepared with or without the addition of ligands were used for EM analysis (Supplementary Figs. 5 and 6). Although cryo-EM analyses of crosslinked TLR7/Cpd-3, TLR7/Cpd-6 and TLR7/Cpd-7 complexes suffered from a severe preferred orientation problem, leading to anisotropic resolution, the resulting 3D reconstitution clearly showed different conformations of TLR7 induced by different ligands (Supplementary Figs. 7–9 and Supplementary Table 2). The TLR7/Cpd-3 complex exclusively adopted a closed-form conformation which is in good agreement with the crystal structure (Fig. 2d, Supplementary Figs. 1 and 7). Surprisingly, the 3D-reconstruction of TLR7/Cpd-6 yielded two major types of TLR7 dimer (Fig. 2e and Supplementary Fig. 8): one was the closed form which was also observed in the crystal structure, and the other was the open form, identified by this study, in which two TLR7 promoters were separated from each other at the bottom of their ring structures. In contrast, Cpd-7 induced only the open form of TLR7, which was well fitted to that observed with the TLR7/Cpd-6 complex (Fig. 2f and Supplementary Fig. 9). Negative-staining electron microscope analyses also showed similar results (Supplementary Fig. 10).

### Visualization of the antagonist binding site

**Fig. 3:**
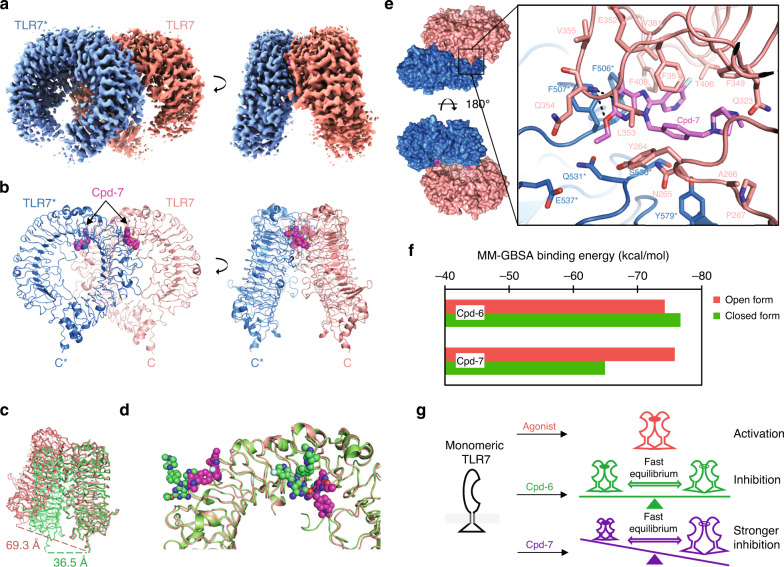
Binding mode of antagonist to the TLR7 open-form structure. **a** Cryo-EM density maps of the TLR7/Cpd-7 complexes resolved by Titan Krios are shown as surface representations. Densities around different TLR7 protomers and Cpd-7 are colored aquamarine, salmon and magenta, respectively. The density map is countered at a level of 0.028. **b** Cryo-EM structure of TLR7/Cpd-7 complex shown in cartoon (TLR7) and space-filling (Cpd-7) representations. The C, O, N and F atoms of Cpd-7 are colored magenta, red, blue and cyan, respectively. **c** Comparison of the open form of the TLR7 dimer (cryo-EM structure of TLR7/Cpd-7 complex; salmon) and the closed form (crystal structure of TLR7/Cpd-6 complex; green). The two structures are superimposed using one protomer. Proteins are shown in ribbon representations. The distances (dashed lines) between the Cα atoms of the two C-terminal residues in both structures are indicated. **d** Comparison of the antagonist binding site in the open-form structure and site 1 in the closed-form structure. The C atoms of Cpd-6 and Cpd-7 are shown in green and magenta, respectively. Two sites are spatially close, but the ligand-binding modes are totally different. **e** Close-up view of Cpd-7 recognition at the antagonist binding site. Top and bottom views of the TLR7/Cpd-7 complex structure in surface representations are shown on the left. Dashed lines indicate hydrogen bonds. **f** Molecular mechanics calculation of the binding energy of Cpd-6 and Cpd-7 for both the open and closed forms. **g** Model of TLR7 ligand action. Unliganded TLR7 is monomeric. Agonists induce the closed form of TLR7 alone, leading to activation. Cpd-6 induced a rapid equilibrium between TLR7 closed and open forms and inhibited TLR7 activation. Cpd-7 predominantly induced the closed form of TLR7, causing stronger inhibition than Cpd-6.

To visualize ligand recognition in the antagonist-bound open-form structure, we determined a high resolution cryo-EM structure of TLR7/Cpd-7 complex at 2.8 Å (Fig. 3a, b, Supplementary Figs. 11 and 12, Supplementary Table 2). We speculated that the inducing of some heterogeneity to the recombinant proteins would help to improve the particle orientation problem. Actually, the distribution of particle orientation was much improved by the use of TLR7 proteins with partially uncleaved purification tags (Supplementary Fig. 12), leading to a high quality cryo-EM map (Supplementary Fig. 13). This TLR7/Cpd-7 complex is also covalently stabilized using glutaraldehyde crosslinker, but no densities of tags and glutaraldehyde were observed. We were able to build a complete model of a C2-symmetric open form of the TLR7 dimer with two molecules of Cpd-7 unambiguously assigned within the protein dimerization interface (Supplementary Fig. 13). The open- and closed-form structures have totally different dimerization interfaces (Supplementary Fig. 14). The distances between the C-termini of two protomers were about 69 Å and 36 Å in the open and closed forms, respectively (Fig. 3c). The larger spatial separation of C-termini in the open form conformation prevents the two intracellular TIR domains from dimerizing and activating TLR7, exhibiting an inhibitory effect.

A defined antagonist binding site is spatially close to the site 1 in the closed form (Fig. 3d), but the binding mechanism differs considerably. TLR7 utilize the common platform for both agonist and antagonist binding on one side of the interface, while the other side is completely different due to a conformational reorganization. Interestingly, although the antagonist was developed based on the agonist, none of the interactions in the recognition of the agonist was conserved in the recognition of antagonist in the open conformation. The recognition of Cpd-7 is mainly characterized by interactions with hydrophobic residues (Fig. 3e). The 6-methyl adenine ring and 8-fluoropyridine moieties were stacked into a channel formed by F408, F506*, F507* (behind Cpd-7) and F349, F351, L353 (in front of Cpd-7) and V381 (on top of Cpd-7). The N1 atom formed a hydrogen bond with Q354 in the main chain. Moreover, the bulky 9-substituent made additional extensive contacts with Q323, F349, Y264, N265, F351, S530* and Y579*.

### Mechanism of TLR7 ligand action

We conducted a molecular mechanics calculation of the binding energy of each ligand to both the open and closed forms to further understand the conformational selectivity of TLR7 induced by different ligands. Three agonists Cpd-1, Cpd-2, and Cpd-3 had higher binding energy in the closed form than the open form (Supplementary Fig. 15). Antagonist Cpd-6 had comparable binding energy in both the open and closed forms, while Cpd-7 showed higher binding energy to the open form (Fig. 3f). These results are consistent with the cryo-EM observations (Fig. 2e, f).

Based on our structural analyses and the results of biomedical and in silico studies, we can summarize the mechanism of TLR7 ligand action (Fig. 3g). Cpd-1, Cpd-2, Cpd-3 and other known TLR7 agonists induce a closed form structure which activates TLR7. The antagonist Cpd-6 induces TLR7 conformational dynamics between the open and closed forms, and inhibits TLR7 activation. The more potent antagonist Cpd-7 predominantly or exclusively induces an open form structure in TLR7 and displays stronger inhibition than Cpd-6.

### Efficacy of Cpd-7 in treating lupus-prone NZB/W F1 mice

**Fig. 4:**
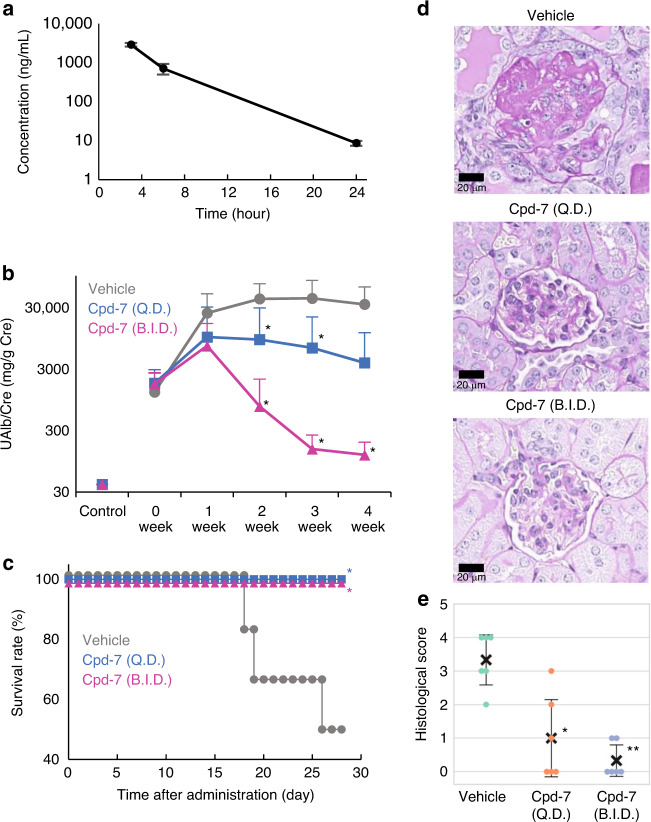
Cpd-7 ameliorates autoimmunity in NZB/W F1 mice. **a** Pharmacokinetics of Cpd-7 following oral administration of a single 100 mg kg^−1^ dose in NZB/W F1 mice. Data are mean ± s.d. (*n* = 3). **b** Time course of proteinuria with oral administration of 100 mg kg^−1^ of Cpd-7 (once or twice daily) or vehicle in NZB/W F1 mice. Data are mean ± s.d. (*n* = 6 per group). *P*-values calculated using Dunnett’s test (two-sided) vs. vehicle. The results were as follows; 2 wk: Q.D. *P* = 0.0375, B.I.D. *P* = 0.0494; 3 wk: Q.D. *P* = 0.0494, B.I.D. *P* = 0.0154; 4 wk: B.I.D. *P* = 0.0068. **c** Survival rate of mice treated in **b** with 100 mg kg^−1^ of Cpd-7 (once or twice daily), or vehicle (*n* = 6 per group). *P*-values calculated using the log-rank test (two-sided) vs. vehicle. The results were as follows; Q.D. *P* = 0.0431, B.I.D. *P* = 0.0431. **d** Histopathological images of the glomeruli with periodic acid-Schiff (PAS) stain. Vehicle control (upper image), group given Cpd-7 at 100 mg kg^−1^ once daily (center image), and group given Cpd-7 at 100 mg kg^−1^ twice daily (lower image). Images are representative PAS stain images with every group. **e** Glomerular lesion score of the group given Cpd-7 100 mg kg^−1^ (once or twice daily) or vehicle. Dot plots of the data with mean ± s.d. (*n* = 6 per group) are shown. *P*-values calculated using Tukey’s test (two-sided) vs. vehicle. The results were as follows; Q.D. *P* = 0.0361, B.I.D. *P* = 0.0043.

Finally, we evaluated the efficacy of Cpd-7 in treating lupus-prone NZB/W F1 mice (Fig. 4a). We verified the pharmacokinetic profile of Cpd-7 in NZB/W F1 mice following oral administration of a single 100 mg kg^−1^ dose, in addition to its inhibitory activity against mouse TLR7 and TLR9 (Fig. 4a and Supplementary Fig. 16). These results confirmed that Cpd-7 is an orally available TLR7-selective inhibitor (IC_50_ = 0.192 μM). Preliminary pharmacokinetic simulations predicted that Cpd-7 would maintain an unbound concentration higher than the IC_50_ values for 20 h with twice daily dosing at 100 mg kg^−1^. Next, we assessed the effect of therapeutic interventions. Cpd-7 was administered orally once or twice daily at 100 mg kg^−1^ for four weeks to female NZB/W F1 mice with a urinary albumin/creatinine ratio over 300 mg g^−1^, which marked the onset of glomerulonephritis. Treatment with Cpd-7 resulted in a significant decrease in proteinuria in a dose-dependent manner compared to vehicle control, and prevented death in all mice that received either dose (*P* < 0.05) (Fig. 4b, c). Histological analysis of the kidneys confirmed that both treatment groups exhibited no glomerular lesions, whereas vehicle control displayed glomerulonephritis accompanied by dilated tubules, regenerating tubules, hyaline casts and perivascular infiltration of mononuclear cells. (Fig. 4d). Moreover, proliferation of the mesangium, an increase in glomerular cellularity, capsular adhesions and/or glomerular sclerosis were observed in the affected glomeruli. Consequently, glomerular lesion scores in the administration group were significantly reduced in a dose-dependent manner compared to those in the vehicle control (Fig. 4e). Although no toxicological evaluation was performed in the efficacy study, no clear toxic signs in the body weights or clinical signs were observed in both treatment groups.

## Discussion

To date, various small-molecule TLR7 agonists have been synthesized and many of them are under clinical trials for treating viral diseases such as hepatitis B and influenza and several types of cancer^21^. In contrast, few small-molecule TLR7-specific antagonist has been reported in literature. Since blocking of TLR7 is considered as a promising strategy for treating autoimmune diseases including SLE and psoriasis^22–24^, there is an urgent need for the development of TLR7-specific antagonist. In this study, starting with a TLR7-specific agonist, we successfully developed TLR7-specific antagonists. Cpd-6 and Cpd-7 with IC_50_ at nano-molar concentrations showed much higher inhibitory activity compared to the indirect TLR inhibitor HCQ. These two compounds also displayed high TLR7 selectivity over both TLR8 and TLR9 which can be considered as TLR7-specific antagonists.

Surprisingly, the crystal structure of TLR7/Cpd-6 complex adopted the closed-form conformation and Cpd-6 was observed inside the agonist binding site, while this complex formed both open- and closed-form conformations with comparable particle numbers in cryo-EM analysis. Meanwhile, the stronger antagonist, Cpd-7, induced a dominant open-form conformation in cryo-EM analysis. We speculate that the closed form of TLR7 induced by antagonist is not sufficient for subsequent signal transduction due to the rapid structural equilibrium.

Superimposing of the TLR7 open-form conformation onto the inactive TLR8 dimer structure also revealed that the two TLR7 protomers were apart from each other more than the TLR8 protomers, demonstrating that these open-form conformations exhibit an inhibited form. Both structures showed the similar interface area with ~1300 Å^2^ (Supplementary Fig. 17)^25^.

To solve the structure, we used the protein with partially cleaved tag, and introduced the cross-linking. No densities of tags and glutaraldehyde were observed, probably because the conformation of the tag and crosslinked residues would not be rigid, only part of the proteins contained the tag, and/or all the residues would not be crosslinked. The overall conformation of the partially tag- cleaved TLR7 protein A tag is the same as the tag-cleaved TLR7. Moreover, the TLR7 structures of the protomer are mostly the same both in X-ray crystallography (tag-cleaved, no crosslinking) and cryo-EM (partially-cleaved, cross-linking). Thus, introduction of the crosslinking and the protein A tag has no serious effect on conformations of TLR7.

The agonist-antagonist conversion was achieved through the introduction of a bulky substituent in the C8 position in 8-OAs (Fig. 1a). The effect of this modification can be interpreted in two different ways, leading to a shift in equilibrium toward the open conformation: in one scenario the bulky substituent destabilizes the activated dimer (Fig. 1b), while in the other the formation of extensive contacts with the antagonist binding pocket stabilizes the open conformation (Fig. 3e). Cpd-6 induces both open and closed forms of TLR7 (Fig. 2e), therefore antagonism of Cpd-6 would result from a rapid equilibrium between the two forms (Fig. 3g). Because the 6-amino group in 8-OAs is critical for TLR7 activation by interacting with D555* (Figs. 1b and 2c), as observed in all the agonist-bound TLR7 structures, the substitution of the 6-amino group (in Cpd-6) with a 6-methyl group (in Cpd-7) subverts the binding of Cpd-7 to the closed-form conformation, thus shifting the equilibrium toward the open conformation and thereby conferring a more potent inhibitory activity on Cpd-7.

Finally, we showed that oral administration of Cpd-7 was effective in the treatment of severe autoimmunity in a mouse model, and potentially should be useful in treating SLE. Our work provides a comprehensive analysis of the mechanism of action of TLR7 antagonists and a potential for TLR7-targeted SLE therapy and would be an incentive for future research and development.

## Methods

### Synthesis of the compounds

Cpd-1 (SM-374527), Cpd-2 (SM-360320), Cpd-3 (SM-394830), Cpd-5 (DSR-070543), Cpd-6 (DSR-139293), Cpd-7 (DSR-139970), CU-CPT8m^26^ were synthesized at Sumitomo Dainippon Pharma, Japan as shown in Supplementary Note. These were used as free form or HCl salts.

### Expression and purification of TLR7

The recombinant TLR7 proteins expressed in *Drosophila* S2 cells (Thermo Fisher Scientific) using Monkey (*Macaca mulatta*) TLR7 extracellular domain expression construct with N167Q, N399Q, N488Q and N799Q mutations and cleavable Z-loop and C-terminal protein A tag were prepared and were stored in a buffer containing 10 mM Tris-HCl pH 7.5 and 150 mM NaCl at concentrations of 10–15 mg ml^−1^. Briefly, the purification steps included IgG affinity purification, the Z-loop and the protein A tag cleavage, and gel-filtration chromatography. Glycan-trimmed TLR7 proteins, expressed in the presence of 1.5 mg l^−1^ kifunensine (Glycosyn) and treated by Endo Hf (New England Biolabs) were used for crystallization. Glycan-untrimmed proteins were used in ITC analysis, crosslinking assay, negative-staining EM and cryo-EM (200 kV microscopes) analyses. Glycan-trimmed TLR7 proteins with partially-uncleaved protein A tag were used for cryo-EM analysis using Tatan Krios (Supplementary Fig. 11).

### Crystallization of TLR7 and structure determination

All crystallization experiments were conducted with sitting-drop vapor-diffusion methods at 293 K. For crystallization of TLR7 with Cpd-1, Cpd-2 and Cpd-3, TLR7 proteins were diluted to 7.5 mg ml^−1^ in buffers containing 10 mM Tris-HCl pH 7.5 and 150 mM NaCl, and either 1 mM or saturated concentrations of ligands. The crystallization droplets were made by mixing 0.5 µl protein solution and 1.0 µl reservoir solution (8%~12% (w/v) PEG3350 or PEG8000 (Hampton Research), 0.3–0.375 M ammonium sulfate, 0.075 M sodium citrate pH 5.0 (Hampton), 2.5 mM Tris-HCl pH 7.5 and 0.0375 M NaCl) using mosquito crystal (TTP Labtech). For crystallization of TLR7/Cpd-6 complex, crystals with good-quality diffraction were obtained by using protein solutions containing 10 mg/ml TLR7 and 2.5 mM Cpd-6 and the reservoir solution containing 0.2 M ammonium sulfate, 0.1 M tri-sodium citrate pH 5.6 and 15% (w/v) PEG4000 (PEGs II Suite, E8) (Qiagen).

Diffraction data sets were collected with a wavelength of 1.0000 Å on beamlines PF BL5A or PF-AR NE3A (Ibaraki, Japan) under cryogenic conditions at 100 K. Crystals were soaked into cryoprotectant solutions containing 15–20% glycerol, and were flash cooled in the cryostream or liquid nitrogen. The diffraction datasets were processed with XDS^27^. Phases were determined by molecular replacement method using the program MOLREP^28^. Coordinates of monkey TLR7 in complex with R848 (PDB ID: 5GMH [10.2210/pdb5GMH/pdb]) were used as the search model. Models were refined with stepwise cycles of manual model building using the COOT program^29^ and restrained refinement using REFMAC5 program^30^ and phenix.refine program^31^. The qualities of the final structures were validated with the PDB validation server (http://wwpdb-validation.wwpdb.org/). Coordinates and the structure factor of all data were deposited in the Protein Data Bank. The statistics of the crystallographic data collection and refinement, and deposited PDB IDs were summarized in Table S1. Structure representations were generated in Pymol^32^.

### ITC analysis

ITC experiments (Fig. 2a) were conducted at 298 K in a final buffer condition of ~30 mM citrate-NaOH pH 4.7 or pH 5.7 (Hampton Research), 150 mM NaCl by using a MicroCal iTC200 (Malvern Panalytical). Solutions containing 1 mM Cpd-6 or Cpd-7 were titrated into solutions containing 80 µM TLR7 proteins, using a titration sequence of a single 0.4 µl injection followed by 18 injections, 2 µl each, with 180 s intervals between injections. OriginLab software (OriginLab) was used to analyze the raw ITC data. Thermodynamic parameters were extracted from curve fitting analysis with a single-site binding model.

### Glutaraldehyde crosslinking assay

For glutaraldehyde crosslinking assay (Fig. 2b), proteins were buffer exchanged into a buffer containing 10 mM HEPES-NaOH, pH 8.0, 150 mM NaCl and were concentrated to ~13 mg/ml using Amicon Ultra devices (Merck). Protein concentrations were adjusted to 25 µM using buffers containing 10 mM HEPES-NaOH, pH 7.0, 150 mM NaCl (for neutral condition) or 50 mM citrate-NaOH pH 5.0, 150 mM NaCl (for acidic condition). Cpd-3, Cpd-6 or Cpd-7 were added to final concentrations of 100 or 1000 µM. The reactions were started by adding glutaraldehyde (Wako) to a final concentration of 5 mM (for neutral condition) or 20 mM (for acidic condition), incubated at ambient temperature for 25 min and quenched by adding Tris-HCl pH 7.5 to a final concentration of 100 mM. Crosslinked samples were analyzed by non-reducing SDS-PAGE. Quantitative analysis of the SDS-PAGE gel image was performed with Image-J.

### Crosslinked sample preparation for EM analyses

For preparation of crosslinked TLR7/Cpd-3 complex for negative-staining EM (Supplementary Fig. 10a) and cryo-EM analyses (Fig. 2d), 25 µM TLR7, 1 mM Cpd-3 and 20 mM glutaraldehyde were mixed in 50 mM citrate-NaOH pH 5.0, 150 mM NaCl buffer and reacted for 30 minutes at ambient temperature. The reaction was quenched by adding 100 mM glycine-HCl pH 5.0. The crosslinked sample was further purified using a repacked Superdex 200 Increase column (GE Healthcare) in 10 mM citrate-NaOH, pH 5.0, 150 mM NaCl buffer (Supplementary Fig. 5). The dimer fractions were pooled and concentrated to ~0.6 mg/ml using Amicon Ultra devices (Merck). Samples were flash cooled in liquid nitrogen and stored at −70°C for further use.

For preparation of crosslinked TLR7 (no ligand), TLR7/Cpd-6 and TLR7/Cpd-7 complexes (Supplementary Fig. 5) for negative-staining EM (Supplementary Fig. 10b–d) and cryo-EM analyses (Fig. 2e, f), 25 µM TLR7 in the absence or presence of 2 mM Cpd-6 or Cpd-7 and 20 mM glutaraldehyde were mixed in 50 mM citrate-NaOH pH 5.0, 150 mM NaCl buffer and reacted for 30 min at ambient temperature. The reactions were quenched by adding 100 mM glycine-HCl pH 5.0. The crosslinked samples were further purified using a Superdex 200 Increase column (GE Healthcare) in 10 mM citrate-NaOH, pH 5.0, 150 mM NaCl buffer (Supplementary Fig. 6). The dimer fractions were pooled and concentrated to ~2 mg/ml using Amicon Ultra devices (Merck). Samples were flash cooled in liquid nitrogen and stored at −70°C for further use.

For preparation of crosslinked TLR7/Cpd-7 complex for cryo-EM analysis using Titan Krios (Fig. 3), 30 µM TLR7 with partially-uncleaved protein A tag (Supplementary Fig. 10a, pooled #1), 2 mM Cpd-7 and 22.5 mM glutaraldehyde were mixed in 50 mM citrate-NaOH pH 4.7, 150 mM NaCl buffer and reacted for 30 minutes at ambient temperature. The reactions were quenched by adding 100 mM glycine-HCl pH 5.0. The crosslinked sample was further purified using a Superdex 200 Increase column (GE Healthcare) in 10 mM citrate-NaOH, pH 4.8, 100 mM NaCl buffer (Supplementary Fig. 11d). The dimer fractions were pooled and concentrated to ~1.4 mg/ml using Amicon Ultra devices (Merck). Samples were flash-cooled in liquid nitrogen and stored at −70 °C for further use.

### Negative-staining EM data acquisition and image processing

The crosslinked TLR7/Cpd-3, TLR7 (no ligand), TLR7/Cpd-6 and TLR7/Cpd-7 complexes samples were diluted to 100 nM using 20 mM citrate-NaOH, pH 5.0, 150 mM NaCl buffer. A 3-µl aliquot of samples was adsorbed for 30 s onto glow discharged holey grids coated with thin carbon (ELS-C10 STEM Cu100P, Okenshoji). After blotted off the excess sample, the grid was stained with 3 µl 2% (w/v) uranyl acetate for three times, and finally air-dried. The stained grids were analyzed using a JEM-2010F microscope (JEOL) at the University of Tokyo running at 200 kV equipped with a 4 K×4 K CCD camera. Data collection was operated at 60,000× magnification and −0.5~−1.5-µm defocus range. For each sample, 40 ~60 CCD images with pixel size of 1.5Å were collected. CTF parameters estimation, automated particle picking, and 2 cycles of 2D classification were performed using cisTEM software^33^ (Supplementary Fig. 10).

### Cryo-EM data acquisition

The crosslinked TLR7/Cpd-3, TLR7/Cpd-6, TLR7/Cpd-7 complexes samples for 200 kV microscope experiments (Fig. 2d–f) were diluted to 0.6–0.7 mg/ml using 10 mM citrate-NaOH pH 4.8, 100 mM NaCl buffer. The crosslinked TLR7/Cpd-7 complex with partially-uncleaved protein A tag sample for 300 kV microscope experiments (Fig. 3) was diluted to ~0.8 mg/ml using 10 mM citrate-NaOH pH 4.8, 100 mM NaCl, 1 mM Cpd-7 buffer. A 3-µl aliquot of samples was applied to a glow-discharged Quantifoil grid (R1.2/R1.3 300 mesh, copper or copper/rhodium), blotted for 4–5 s in 100% humidity at 4–6 °C and plunged into liquid ethane using a Vitrobot MkIV (Thermo Fisher Scientific). The Cryo-EM analysis was initially performed by using a Talos Arctica microscope (Thermo Fisher Scientific) at 200 kV with a Falcon3EC direct electron detector at the Cryo-EM facility in KEK (Ibaraki, Japan). Data collections of TLR7/Cpd-3 complex (Fig. 2d and Supplementary Fig. 7) and TLR7/Cpd-6 complex (Fig. 2e and Supplementary Fig. 8) were performed by using a Glacios microscope (Thermo Fisher Scientific) at 200 kV with a Falcon3EC direct electron detector in the counting mode at RIKEN RSC Cryo-EM facility (Hyogo, Japan). Movie stacks were acquired using 150,000× magnification with an accumulated dose of 50.0 electrons per Å^2^ over 40 frames. The pixel size was 0.67 Å. The data were automatically acquired by the beam-image shift method using the EPU software. Data collection of TLR7/Cpd-7 complex (Fig. 2f, Supplementary Fig. 9) was performed by using a Talos Arctica microscope (Thermo Fisher Scientific) at 200 kV equipped with a Gatan Quantum-LS Imaging Filter (GIF) and a Gatan K2 Summit direct electron detector in the electron counting mode at the Cryo-EM facility in the University of Tokyo (Tokyo, Japan). Movie stacks were acquired using 130,000× magnification with an accumulated dose of 50.0 electrons per Å^2^ over 40 frames. The pixel size was 1.03 Å. The data were automatically acquired by the beam-image shift method using the SerialEM software^34^. The high resolution data collection of TLR7/Cpd-7 complex with partially-uncleaved protein A tag sample (Supplementary Fig. 12) was performed by using a Titan Krios G3i microscope (Thermo Fisher Scientific) at 300 kV equipped with a Gatan Quantum-LS Imaging Filter (GIF) and a Gatan K3 direct electron detector in the electron counting mode at the Cryo-EM facility in the University of Tokyo (Tokyo, Japan). Movie stacks were acquired using 105,000× magnification with an accumulated dose of 60.0 electrons per Å^2^ over 60 frames. The pixel size was 0.83 Å. The data were automatically acquired by the beam-image shift method using the SerialEM software^34^.

### Cryo-EM data processing and model building

Cryo-EM data processing were performed using RELION 3.0^35^. Raw movies stacks were motion-corrected using MotionCor2^36^ or RELION’s own implementation^35^. The CTF parameters were determined using the CTFFIND4 program^37^. Data processing workflows for TLR7/Cpd-3 complex (940 movie stacks from Glacios), TLR7/Cpd-6 complex (2,988 movie stacks from Glacios), TLR7/Cpd-7 complex (2,547 movie stacks from Talos Arctica), TLR7/Cpd-7 complex with partially-uncleaved protein A tag (4,464 movie stacks from Titan Krios) are summarized in Supplementary Figs. 6, 7, 8, and 11, respectively. The final resolution was estimated by gold-standard Fourier shell correlation (FSC) between the two independently refined half maps (FSC = 0.143). Because the first three data suffered from severe particle orientation preference which led to anisotropic resolution, we did not build structural models against these maps. In the TLR7/Cpd-6 complex dataset analysis, two distinct forms of TLR7 dimer were gained after the first round of 3D classification and each of them was refined separately (Supplementary Fig. 8). In the Titan Krios data processing, a total of three rounds of 3D classification generated a particle package containing 301,212 particles, which yielded an overall resolution of 2.8 Å (Supplementary Fig. 12). For model building against the EM map, coordinates of two TLR7 monomers (PDB ID: 5GMH [10.2210/pdb5GMH/pdb]) were docked into the final map using Chimera^38^. The model was then manually refined using the COOT program^29^. Real-space refinement in Phenix program^39^ was performed after manual refinement.

The cryo-EM maps have been deposited in the Electron Microscopy Data Bank. The atomic coordinates of the TLR7/Cpd-7 complex structure have been deposited in the Protein Data Bank. The statistics of EM data processing and refinement were summarized in Supplementary Table 2. The local resolution maps were produced with the ResMap program^40^. Structure representations were generated in the Chimera^38^, ChimeraX^41^, Pymol^32^ and COOT^29^ programs.

### Binding energy calculations

To calculate binding energies of Cpd-1, Cpd-2, Cpd-3, Cpd-6 and Cpd-7 to TLR7, the complex structures (TLR7Cpd-6 complex (closed form, crystal structure) and TLR7/Cpd-7 complex (open form, cryo-EM structure)) were processed using the protein preparation wizard^42^ in Maestro^43^. Structures of compounds were modeled from the complex structures. Then, all complex structures were minimized using the OPLS3e force field^44^. The binding energies of Cpd-1, Cpd-2, Cpd-3, Cpd-6 and Cpd-7 to open and closed forms of TLR7 were calculated using the MM-GBSA method^45^ with the VSGB solvation model^46^ and the OPLS3e force field^44^.

### Mice

Female NZBWF1/Slc (NZBW F1) mice over 25 weeks of age (Japan SLC Inc., Japan) were used in this study. All mice were maintained under specific pathogen-free conditions. All experiments were approved by the Institutional Animal Care and Use Committee at Sumitomo Dainippon Pharma.

### Pharmacokinetic profiling

Cpd-7 was orally administered to 35-week-old female NZB/W F1 mice at 100 mg kg^−1^ dissolved in 0.5 w/v% Methyl Cellulose 400 Solution (WAKO, Japan). Blood was collected over 24 h from the isoflurane-anesthetized mice via the jugular vein. The plasma samples were separated by centrifugation. Cpd-7 was extracted from the plasma samples by protein precipitation with methanol and measured using LC/MS/MS (HPLC: SHIMADZU CORPORATION, Japan; MS: AB Sciex Pte. Ltd., Japan). The lower limit of quantification was set at 5 ng/mL.

### Animal study

Cpd-7 was dissolved in 0.5 w/v% Methyl Cellulose 400 Solution (WAKO) at 10 mg/ml. NZBW F1 mice were orally treated with Cpd-7 for 4 weeks. At the initiation of treatment, mice were randomly divided according to bodyweight and proteinuria level (urinary albumin/creatinine ratio over 300 mg g^−1^) and were orally once or twice daily. In treatment, mice were divided into 3 groups (*n* = 6 per group): vehicle control group (Methyl Cellulose 400 Solution, 10 µl g^−1^ body weight of mice), Cpd-7 once treated group (Q.D.: 100 mg kg^−1^, day), and Cpd-7 twice treated group (B.I.D.: 100 mg kg^−1^, twice day), and the bodyweight and proteinuria level were monitored weekly. During the treatment, nearly dead individuals were euthanized, then sera and kidneys were collected. In this case, the last known values for urinary protein was carried forward. No clear toxic signs in the body weights or clinical signs were observed in both treatment groups.

### Quantification of urine albumin

Urine protein was quantified by automatic analyzer (BioMajesty ZERO(JCA-ZS050), JEOL Ltd, Japan) with Mouse Urinary Albumin Assay Kit (S-type) (AKRAL-021S, Shibayagi Co.,Ltd., Japan). Urine creatinine levels were measured in the same samples. The urine albumin excretion rate is expressed as the ratio of albumin to creatinine. Time course of proteinuria were analyzed by Dunnett’s multiple-range test.

### Histopathological analysis

The both kidneys were fixed in 10% neutral-buffered formalin, embedded in paraffin, sectioned, stained with hematoxylin and eosin (HE) and periodic acid-Schiff (PAS), and examined by light microscopy. Glomerulonephritis was scored on a 0–4 scale, based on the intensity and extent of histopathological changes^47^. A grade of 0 was given to kidney without glomerular lesions; a grade of 1 was given when there was minimal thickening of the mesangium; a grade of 2 was given when there was a noticeable increase in both mesangial and glomerular capillary cellularity; a grade of 3 was given when the preceding conditions were observed, along with superimposed inflammatory exudates and capsular adhesions; and a grade of 4 was given when the obliteration of the glomerular architecture included 70% of glomeruli. These classifications were used to grade 20 glomeruli within one area, and the mean glomerular histopathological score was calculated for each mouse. Data were analyzed by Tukey’s test.

### Human TLR reporter gene inhibitory activity assays

Compounds were dissolved in dimethyl sulfoxide. Before stimulation with compound, Human TLR7 reporter cell line (TLR7/NF-κB/SEAPorter^TM^ HEK293 cell, Novus Biological, USA), human TLR8 reporter cell line (TLR8/NF-κB/SEAPorter^TM^ HEK293 cell, Novus Biological), and human TLR9 reporter cell line (TLR9/NF-κB/SEAPorter^TM^ HEK293 cell, Novus Biological) were seeded in 96-well microtiter plate (AGC techno glass, Japan) at 5 × 10^4^ cells/well in DMEM (GIBCO, United Kingdam) with 10 % fetal bovine serum (GIBCO), and 10 ug/ml Blastcidin S HCl (GIBCO) and incubated at 37 °C in 5% CO_2_. The plates were incubated over-night and then stimulated with various concentrations of compound. HCQ (human TLR7/9, Acros Organics, United Kingdom) and CU-CPT8m (human TLR8, synthesized at Sumitomo Dainippon Pharma, Japan) were used as positive control. After 30 min, R848 (human TLR7/8 ligand, synthesized at Sumitomo Dainippon Pharma, Japan) or CpG2006 (human TLR9 ligand, Hokkaido System Science Co., Ltd., Japan) added to each well, wherein each final concentration were adjusted to 200 nM R848 (human TLR7), 30 μM R848 (human TLR8), or 500 nM CpG2006 (human TLR9). The plates were then incubated an additional 20 ± 1 h at 37 °C in 5% CO_2_ and then the secreted embryonic alkaline phosphatase (SEAP) activity was measured as activation of human TLR7, 8, and 9. The SEAP activity was evaluated as follows: pNPP (Invitrogen, USA) was added to the incubated sample (50 μl/well); after 15 min, 4 M sodium hydroxide solution (nacalai tesque, Japan) was added thereto (50 μl/well) to quench the reaction; and the absorbance of each sample was measured at 405 nm with microplate reader SpectraMax M4 (Molecular Devices, USA). The 50 % inhibitory concentration (IC_50_ value) of each compound was calculated based on 100 % of the SEAP activity wherein the test sample comprises no compound.

### Human TLR7 reporter gene agonistic activity assays

Compounds were dissolved in dimethyl sulfoxide. Before stimulation with compound, Human TLR7 reporter cell line (Novus Biological) was seeded in 96-well microtiter plate (AGC techno glass) at 5 × 10^4^ cells/well in DMEM (GIBCO) with 10% fetal bovine serum (GIBCO), and 10 ug/ml Blastcidin S HCl (GIBCO) and incubated at 37 °C in 5% CO_2_. The plates were incubated over-night and then stimulated with various concentrations of compound and R848. The plates were then incubated an additional 20 ± 1 h at 37 °C in 5% CO_2_ and then the secreted embryonic alkaline phosphatase (SEAP) activity was measured as activation of human TLR7. The SEAP activity was evaluated as follows: pNPP (Invitrogen, USA) was added to the incubated sample (50 μl/well); after 15 min, 4 M sodium hydroxide solution (nacalai tesque, Japan) was added thereto (50 μl/well) to quench the reaction; and the absorbance of each sample was measured at 405 nm with microplate reader SpectraMax M4 (Molecular Devices, USA). The 50 % effective concentration (EC_50_ value) of each compound was calculated based on 100 % of the SEAP activity wherein the test sample comprises no compound.

### Cytokine measurement using PBMC

Human Peripheral Blood Mononuclear cells (hPBMCs) were collected from healthy adult donors. hPBMCs were cultured in round-bottom 96-well plates (BD Falcon, Durham, NC, USA) with a density of 5 × 10^5^ cells/well. Plated cells were pretreated with DMSO or 2.5 μM inhibitors for 3 h and then stimulated by indicated ligands, such as R848 or TLR1/2 specific ligand Pam_3_CSK_4_ (Invivogen, HongKong, China), for 20 h in 200 ml of RPMI 1640 medium (Gibco, Paisley, UK) supplemented with 10% FBS, Penicillin-Streptomycin-Glutamine (Gibco, Paisley, UK), 50 μM 2-ME medium at 37 °C in the presence of 5% CO_2_. Supernatants were collected to measure secreted cytokines by ELISA. Concentration of human IFN-α and TNF-α in the supernatants was measured with Verikine Human IFN-α Multi-Subtype Serum ELISA Kit (PBL Assay Science, Piscataway, NJ, USA) and Human TNF-α Uncoated ELISA (ThermoFisher Scientific, Carlsbad, CA, USA), respectively. This study was approved by the Research Ethics Committee of IMSUT (accession number: 30-100-B20190419).

### Analyses of dose-response relationship

Compounds were dissolved in dimethyl sulfoxide. Human TLR7 reporter cell line (Novus Biological) were seeded in 96-well microtiter plate (AGC techno glass) at 5 × 10^4^ cells/well in DMEM (GIBCO) with 10% fetal bovine serum (GIBCO), and 10 μg ml^−1^ Blastcidin S HCl (GIBCO) and incubated at 37 °C in 5% CO_2_. The plates were incubated over-night and then addition with various concentrations of compound. After 30 min, R848 (human TLR7/8 ligand) that was diluted with the medium was added, wherein final concentration was adjusted to 0.01, 0.03, 0.1, 0.3, 1, 3, 10, 30, 100, 300 and 1000 μM. The plates were then incubated an additional 20 ± 1 h at 37 °C in 5% CO_2_, and then the SEAP activity was measured as activation of human TLR7. The SEAP activity was evaluated as follows: pNPP (Invitrogen) was added to the incubated sample (50 μl/well); after 15 min, 4 mol l^−1^ sodium hydroxide solution (nacalai tesque) was added thereto (50 μl/well) to quench the reaction; and the absorbance of each sample was measured at 405 nm with microplate reader SpectraMax M4 (Molecular Devices).

### Mouse TLR reporter gene assays

Compounds were dissolved in dimethyl sulfoxide. The day before transfection, mouse TLR7 genestably-expressing HE293 cell line (293XL-mTLR7 cell, InvivoGen, USA) and mouse TLR9 genestably-expressing HEK293 cell line (293-mTLR9 cell, InvivoGen) were seeded in collagen-coated 6-well plate (AGC techno glass) at 3 × 10^5^ cells/well in DMEM (GIBCO) with 10% fetal bovine serum (GIBCO), and 10 μg ml^−1^ Blastcidin S HCl (GIBCO) and incubated at 37 °C in 5% CO_2_. The plates were incubated over-night and then cotransfected with 1 μg (mouse TLR7) or 0.3 μg (mouse TLR9) of pNF-kB-Luciferase reporter in a 10:1 ratio with 4 μl of FuGene6 transfection reagent (Promega corporation, USA) following the manufacturer’s instructions. The plates were incubated 24 h. The cells re-seeded into 96-well plate (AGC techno glass) at 2 × 10^4^ cells/well in DMEM (GIBCO) with 10% fetal bovine serum (GIBCO), and 10 μg ml^−1^ Blastcidin S HCl (GIBCO) and then inhibition with various concentrations of compound. HCQ (mouse TLR7/9, Acros Organics, United Kingdom) was used as positive control. After 30 min, R848 (mouse TLR7/8 ligand) or CpG1826 (mouse TLR9 ligand, Hokkaido System Science Co., Ltd.) added to each well, wherein each final concentration were adjusted to 200 nM R848 (mouse TLR7) or 100 nM CpG1826 (mouse TLR9). The plates were then incubated an additional 6 h at 37 °C in 5% CO_2_, and then the luciferase activity was measured as activation of mouse TLR7 and 9. The luciferase activity was evaluated as follows: Bright-GloTM Luciferase Assay System (Promega) was added to the incubated sample (100 μl/well); after 2 min, the luminescence intensity of each sample was measured with a luminometer (Envision). The IC_50_ value of each sample compound was calculated based on 100 % of the luciferase activity wherein the test sample comprises no sample compound.

### Solubility measurements

Compound was dissolved in dimethyl sulfoxide at 10 mM. A 1.75% disodium hydrogen phosphate aqueous solution and a 5.53% citric acid aqueous solution were mixed while monitoring with a pH meter to prepare an isotonic buffer solution having a pH of 7.4. The dimethyl sulfoxide solution and the isotonic buffer solution of pH 7.4 was mixed and shaken at 110 rpm for 90 min at 25 °C by a constant temperature incubator shaker. After standing for 16–20 h, the mixture was centrifuged to precipitate insoluble matter, and the supernatant was collected. Separately, standard solutions of the compound were prepared by dissolving the compound in a mixture of acetonitrile and water (1:1). The analytical sample and the standard solutions were measured by UHPLC-UV (Waters, USA). The degree of solubility was calculated by comparing the peak area of the analytical sample with standard solutions.

### Reporting summary

Further information on research design is available in the Nature Research Reporting Summary linked to this article.

## Supplementary information

Supplementary Information

Reporting Summary

## Data Availability

The cryo-EM maps are deposited in the Electron Microscopy Data Bank under accession codes EMD-0999 (TLR7/Cpd-3 complex), EMD-1000 (TLR7/Cpd-6 complex in closed form), EMD-30000 (TLR7/Cpd-6 complex in open form), EMD-30001 (TLR7/Cpd-7 complex, Talos data) and EMD-30002 (TLR7/Cpd-7 complex, Krios data). Structure coordinates are deposited at the Protein Data Bank with accession codes 6LVX [10.2210/pdb6LVX/pdb] (TLR7/Cpd-1 complex), 6LVY [10.2210/pdb6LVY/pdb] (TLR7/Cpd-2 complex), 6LVZ [10.2210/pdb6LVZ/pdb] (TLR7/Cpd-3 complex), 6LW0 [10.2210/pdb6LW0/pdb] (TLR7/Cpd-6 complex) and 6LW1 [10.2210/pdb6LW1/pdb] (TLR7/Cpd-7 complex, Krios data). All data needed to evaluate the conclusions in the paper are present in the paper and/or the Supplementary Materials and Source Data. Additional data related to this paper may be requested from the authors.

## Source data

Source Data File

## References

[1] Barchet W (2005). Dendritic cells respond to influenza virus through TLR7- and PKR-independent pathways. Eur. J. Immunol..

[2] Diebold SS, Kaisho T, Hemmi H, Akira S, e Sousa CR (2004). Innate antiviral responses by means of TLR7-mediated recognition of single-stranded RNA. Science.

[3] Zhang Z (2016). Structural analysis reveals that toll-like receptor 7 is a dual receptor for guanosine and single-stranded RNA. Immunity.

[4] Zhang Z (2018). Structural analyses of toll-like receptor 7 reveal detailed RNA sequence specificity and recognition mechanism of agonistic ligands. Cell Rep..

[5] Kim YM, Brinkmann MM, Paquet ME, Ploegh HL (2008). UNC93B1 delivers nucleotide-sensing toll-like receptors to endolysosomes. Nature.

[6] Lee BL, Barton GM (2014). Trafficking of endosomal Toll-like receptors. Trends Cell Biol..

[7] Deane JA (2007). Control of toll-like receptor 7 expression is essential to restrict autoimmunity and dendritic cell proliferation. Immunity.

[8] Fairhurst AM (2008). Yaa autoimmune phenotypes are conferred by overexpression of TLR7. Eur. J. Immunol..

[9] Pisitkun P (2006). Autoreactive B cell responses to RNA-related antigens due to TLR7 gene duplication. Science.

[10] Souyris M (2018). TLR7 escapes X chromosome inactivation in immune cells. Sci. Immunol..

[11] Desnues B (2014). TLR8 on dendritic cells and TLR9 on B cells restrain TLR7-mediated spontaneous autoimmunity in C57BL/6 mice. Proc. Natl Acad. Sci. USA.

[12] Santiago-Raber M-L, Baudino L, Izui S (2009). Emerging roles of TLR7 and TLR9 in murine SLE. J. Autoimmun..

[13] Robbins M (2007). 2′-O-methyl-modified RNAs act as TLR7 antagonists. Mol. Ther..

[14] Wallace DJ, Gudsoorkar VS, Weisman MH, Venuturupalli SR (2012). New insights into mechanisms of therapeutic effects of antimalarial agents in SLE.. Nat. Rev. Rheumatol..

[15] Hirota K (2002). Discovery of 8-hydroxyadenines as a novel type of interferon inducer. J. Med. Chem..

[16] Koga-Yamakawa E (2013). Intratracheal and oral administration of SM‐276001: a selective TLR7 agonist, leads to antitumor efficacy in primary and metastatic models of cancer. Int. J. Cancer.

[17] Kurimoto A (2010). Synthesis and biological evaluation of 8-oxoadenine derivatives as toll-like receptor 7 agonists introducing the antedrug concept. J. Med. Chem..

[18] Nakamura T (2013). Synthesis and evaluation of 8-oxoadenine derivatives as potent Toll-like receptor 7 agonists with high water solubility. Bioorg. Med. Chem. Lett..

[19] Hirota K, Kazaoka K, Sajiki H (2003). Synthesis and biological evaluation of 2, 8-disubstituted 9-benzyladenines: discovery of 8-mercaptoadenines as potent interferon-inducers. Bioorg. Med. Chem..

[20] Kawai T, Akira S (2006). Innate immune recognition of viral infection. Nat. Immunol..

[21] Patinote C (2020). Agonist and antagonist ligands of toll-like receptors 7 and 8: Ingenious tools for therapeutic purposes. Eur. J. Med. Chem..

[22] Zhu F-G (2013). A novel antagonist of Toll-like receptors 7, 8 and 9 suppresses lupus disease-associated parameters in NZBW/F1 mice. Autoimmunity.

[23] Jiang W (2013). A Toll-like receptor 7, 8, and 9 antagonist inhibits Th1 and Th17 responses and inflammasome activation in a model of IL-23-induced psoriasis. J. Investig. Dermatol..

[24] Kanno A (2015). Targeting cell surface TLR7 for therapeutic intervention in autoimmune diseases. Nat. Commun..

[25] Tanji H, Ohto U, Shibata T, Miyake K, Shimizu T (2013). Structural reorganization of the Toll-like receptor 8 dimer induced by agonistic ligands. Science.

[26] Zhang S (2018). Small-molecule inhibition of TLR8 through stabilization of its resting state. Nat. Chem. Biol..

[27] Kabsch W (2010). XDS. Acta Crystallogr. Sect. D Biol. Crystallogr..

[28] Vagin A, Teplyakov A (1997). MOLREP: an automated program for molecular replacement. J. Appl. Crystallogr..

[29] Emsley P, Cowtan K (2004). Coot: model-building tools for molecular graphics. Acta Crystallogr. Sect. D Biol. Crystallogr..

[30] Murshudov GN (2011). REFMAC5 for the refinement of macromolecular crystal structures. Acta Crystallogr. Sect. D Biol. Crystallogr..

[31] Afonine PV (2012). Towards automated crystallographic structure refinement with phenix. refine. Acta Crystallogr. Sect. D Biol. Crystallogr..

[32] DeLano WL (2002). Pymol: An open-source molecular graphics tool. CCP4 Newsl. Protein Crystallogr..

[33] Grant T, Rohou A, Grigorieff N (2018). cisTEM, user-friendly software for single-particle image processing. Elife.

[34] Mastronarde DN (2003). SerialEM: a program for automated tilt series acquisition on Tecnai microscopes using prediction of specimen position. Microsc. Microanal..

[35] Zivanov J (2018). New tools for automated high-resolution cryo-EM structure determination in RELION-3. Elife.

[36] Zheng SQ (2017). MotionCor2: anisotropic correction of beam-induced motion for improved cryo-electron microscopy. Nat. Methods.

[37] Rohou A, Grigorieff N (2015). CTFFIND4: Fast and accurate defocus estimation from electron micrographs. J. Struct. Biol..

[38] Pettersen EF (2004). UCSF Chimera—a visualization system for exploratory research and analysis. J. Comput. Chem..

[39] Adams PD (2010). PHENIX: a comprehensive Python-based system for macromolecular structure solution. Acta Crystallogr. Sect. D Biol. Crystallogr..

[40] Kucukelbir A, Sigworth FJ, Tagare HD (2014). Quantifying the local resolution of cryo-EM density maps. Nat. Methods.

[41] Goddard TD (2018). UCSF ChimeraX: meeting modern challenges in visualization and analysis. Protein Sci..

[42] Sastry GM, Adzhigirey M, Day T, Annabhimoju R, Sherman W (2013). Protein and ligand preparation: parameters, protocols, and influence on virtual screening enrichments. J. Computer Aided Mol. Des..

[43] Linares LK (2007). Intrinsic ubiquitination activity of PCAF controls the stability of the oncoprotein Hdm2. Nat. Cell Biol..

[44] Roos K (2019). OPLS3e: extending force field coverage for drug-like small molecules. J. Chem. Theory Comput..

[45] Papai G (2020). Structure of SAGA and mechanism of TBP deposition on gene promoters. Nature.

[46] Li J (2011). The VSGB 2.0 model: a next generation energy model for high resolution protein structure modeling. Proteins Struct. Funct. Bioinforma..

[47] Wang B, Yamamoto Y, El-Badri NS, Good RA (1999). Effective treatment of autoimmune disease and progressive renal disease by mixed bone-marrow transplantation that establishes a stable mixed chimerism in BXSB recipient mice. Proc. Natl Acad. Sci. USA.

