# Supplementary material for: Structural analysis reveals TLR7 dynamics underlying antagonism
Source: Nat Commun. 2020 Oct 15;11:5204. doi: 10.1038/s41467-020-19025-z (PMC7562955; doi:10.1038/s41467-020-19025-z)
Supplement: Supplementary file 1 — Supplementary Information [file 41467_2020_19025_MOESM1_ESM.pdf]

Supplementary information

Structural analysis reveals TLR7 dynamics underlying antagonism

Shingo Tojo[*†1], Zhikuan Zhang[*2], Hiroyuki Matsui[1], Masahiro Tahara[1], Mitsunori Ikeguchi[3], Mami Kochi[1], Mami Kamada[1], Hideki Shigematsu[4], Akihisa Tsutsumi[5], Naruhiko Adachi[6], Takuma Shibata[7], Masaki Yamamoto[4], Masahide Kikkawa[5], Toshiya Senda[6], Yoshiaki Isobe[1], Umeharu Ohto[†2] and Toshiyuki Shimizu[†2]

Affiliations

[1]Sumitomo Dainippon Pharma Co., Ltd., 3-1-98 Kasugade-naka, Konohana-ku, Osaka 554-0022, Japan

[2]Graduate School of Pharmaceutical Sciences, The University of Tokyo, 7-3-1 Hongo, Bunkyo-ku, Tokyo 113-0033, Japan

[3]Computational Life Science Laboratory, Graduate School of Medical Life Science, Yokohama City University, 1-7-29, Suehiro-cho, Tsurumi-ku, Yokohama, Kanagawa 230-0045, Japan

[4]RIKEN SPring-8 Center, 1-1-1 Kouto, Sayo, Hyogo 679-5148, Japan

[5]Department of Cell Biology and Anatomy, Graduate School of Medicine, the University of Tokyo, 7-3-1 Hongo, Bunkyo-ku, Tokyo 113-0033, Japan

[6]Structural Biology Research Center, Institute of Materials Structure Science, High Energy Accelerator Research Organization (KEK), 1-1 Oho, Tsukuba, Ibaraki 305-0801, Japan

[7] Division of Innate Immunity, Department of Microbiology and Immunology, The Institute of Medical Science, The University of Tokyo, 4-6-1 Shirokanedai, Minato-ku, Tokyo 108-8639, Japan

†Correspondence to: shingo-tojo@ds-pharma.co.jp (S.T.); umeji@mol.f.u-tokyo.ac.jp (U.O.); shimizu@mol.f.u-tokyo.ac.jp (T.Sh.)

*These authors contributed equally to this work.

This file includes:

1. Supplementary Figs. 1 to 17
2. Supplementary Tables 1 to 2
3. Synthetic procedures and characterization (including references)

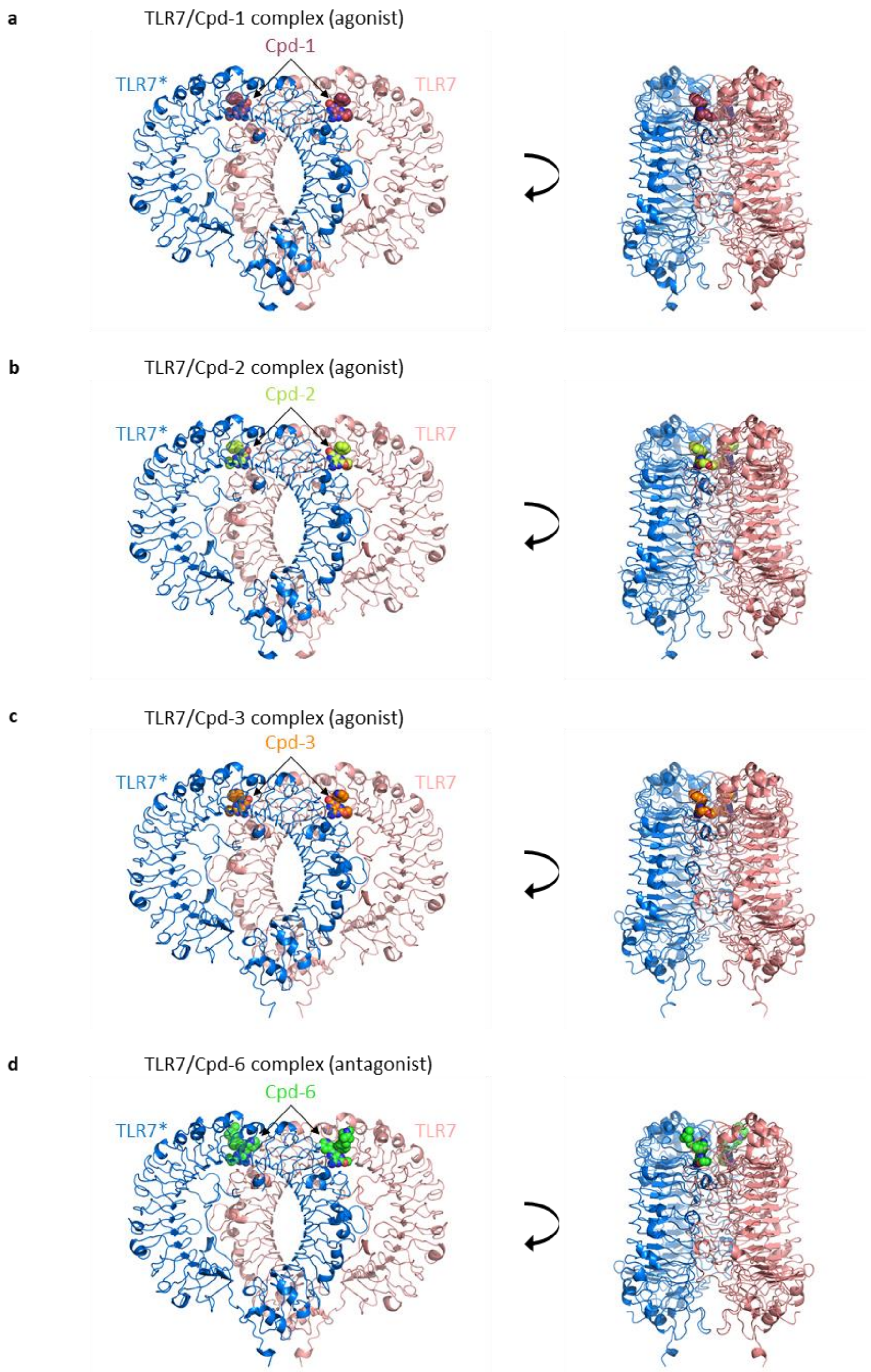

Supplementary Fig. 1. Crystal structures of TLR7/ligand complexes

a-d) Crystal structures of TLR7/Cpd-1, TLR7/Cpd-2, TLR7/Cpd-3 and TLR7/Cpd-6 complexes. The overall structures of the front and side views are shown in cartoon representations, respectively on the left and right. Ligands are shown in space-filling representations. The C atoms of Cpd-1, Cpd-2, Cpd-3 and Cpd-6 have different colors.

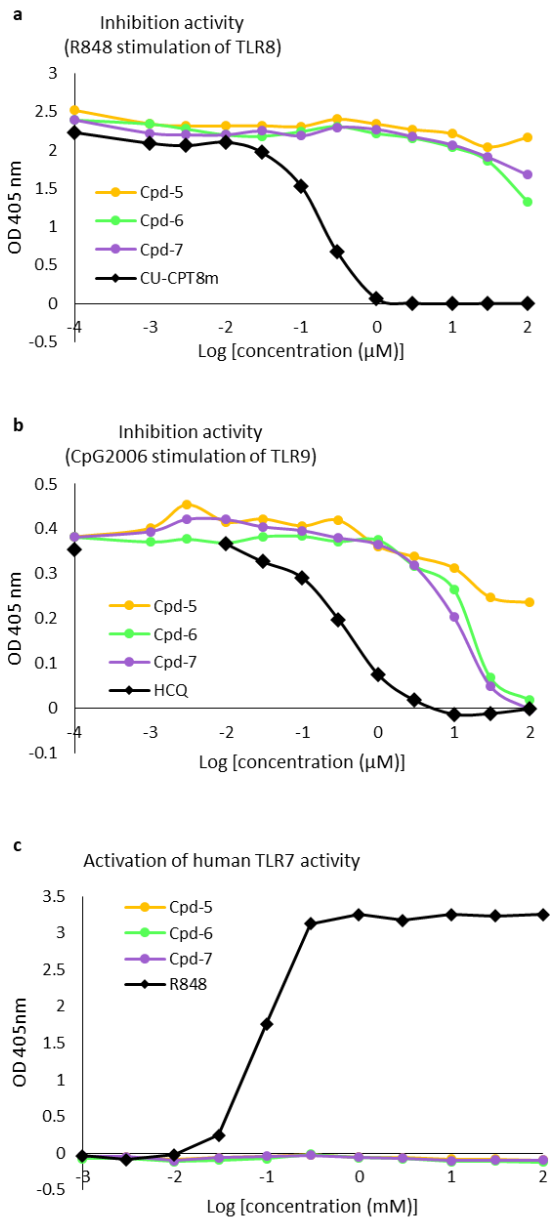

Supplementary Fig. 2. Selective inhibition of TLR7 by Cpd-5, Cpd-6 and Cpd-7

a) Dose-response curves for the inhibition of human TLR8 activity by Cpd-5, Cpd-6, Cpd-7, and CU-CPT8m (positive control) measured by reporter gene assay with R848 (30 mM) as stimulating agent. Data are representative tests with three replicates. **b)** Dose-response curves for the inhibition of human TLR9 activity of Cpd-5, Cpd-6, Cpd-7, and HCQ (positive control) measured by reporter gene assay with CpG2006 (500 nM) as stimulating agent. Data are representative tests with three replicates. **c)** Dose-response curves for the activation of human TLR7 activity by Cpd-5, Cpd-6, Cpd-7 and R848 (positive control) measured by reporter gene assay. Data are representative tests with three replicates.

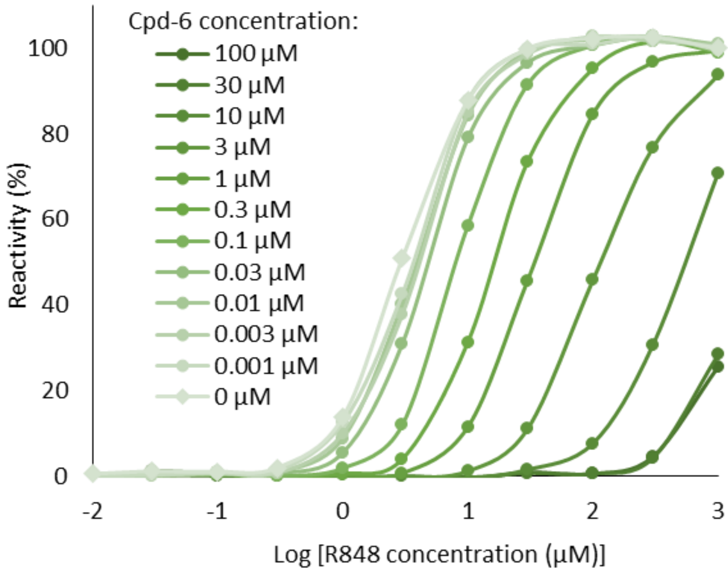

Supplementary Fig. 3. Cpd-6 competitively inhibit R848 activation of TLR7

Dose-response relationship between Cpd-6 and R848 measured by human TLR7 reporter gene assay. Data are representative tests with three replicates.

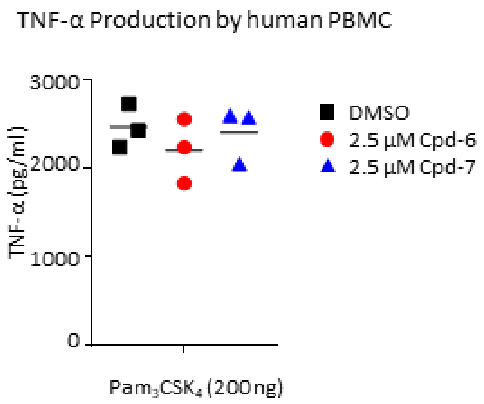

Supplementary Fig. 4. TNF-α production by human PBMC stimulated by TLR1/2 ligand

PBMCs were pretreated by DMSO, Cpd-6 or Cpd-7 for 3 hours before 20-hour stimulation by PAM_3CSK_4. TNF-α levels quantified by ELISA were shown. Dot plots of the data with mean ± s.d. (n=3 per group) are shown.

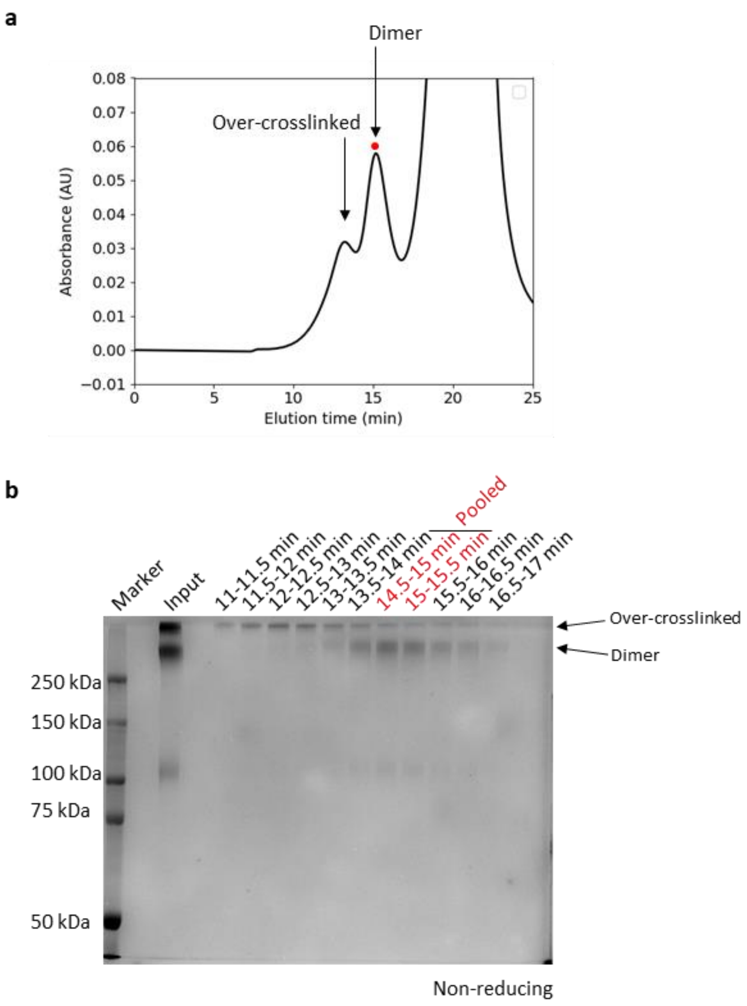

Supplementary Fig. 5. Preparation of crosslinked TLR7/Cpd-3 complex

a) Purification of crosslinked TLR7/Cpd-3 complex by gel filtration chromatography after glutaraldehyde crosslinking. Absorbance at 280 nm is indicated by solid lines. The red dot indicates the peak with subsequent pooling for further use. **b)** SDS-PAGE analysis (non-reducing) of the eluates of the gel filtration chromatography. The pooled fractions are indicated in red. The SDS-page is a representative image with one experiment.

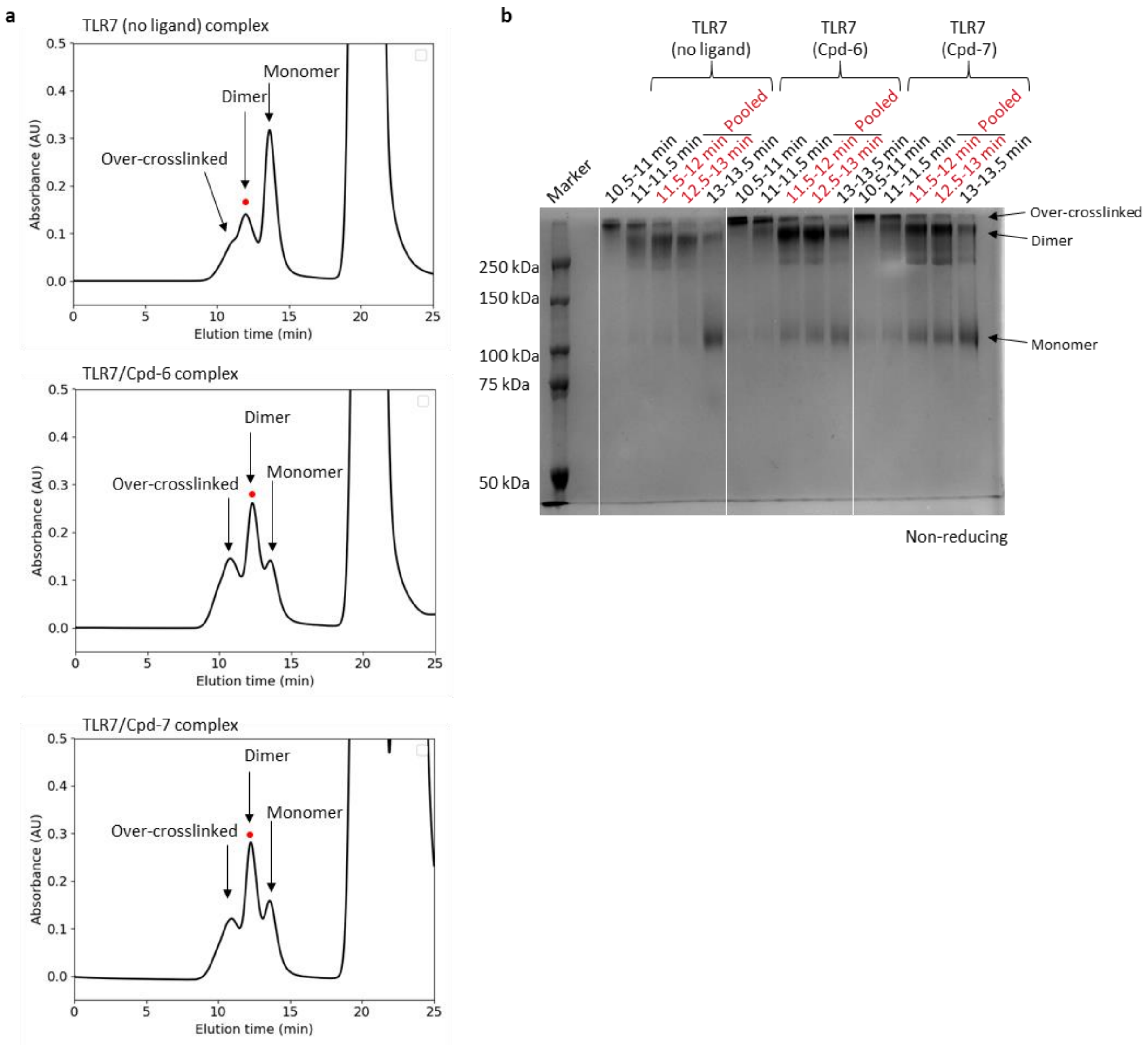

Supplementary Fig. 6. Preparation of crosslinked TLR7 (no ligand), TLR7/Cpd-6 and TLR7/Cpd-7 complexes

a) Purification of crosslinked TLR7 (no ligand), TLR7/Cpd-6 and TLR7/Cpd-7 complexes by gel filtration chromatography after glutaraldehyde crosslinking. Absorbance at 280 nm is indicated by solid lines. The red dot indicates the peak with subsequent pooling for further use. In the absence of ligand, TLR7 could still be crosslinked as a dimer, which might be due to non-specific interactions (top panel). Nonetheless, the amount of ligand-independent dimer was much lower than was the case with Cpd-6 and Cpd-7 (middle and bottom panels). **b)** SDS-PAGE analysis (non-reducing) of the eluates of the gel filtration chromatography. The pooled fractions are indicated in red. The SDS-page is a representative image with one experiment.

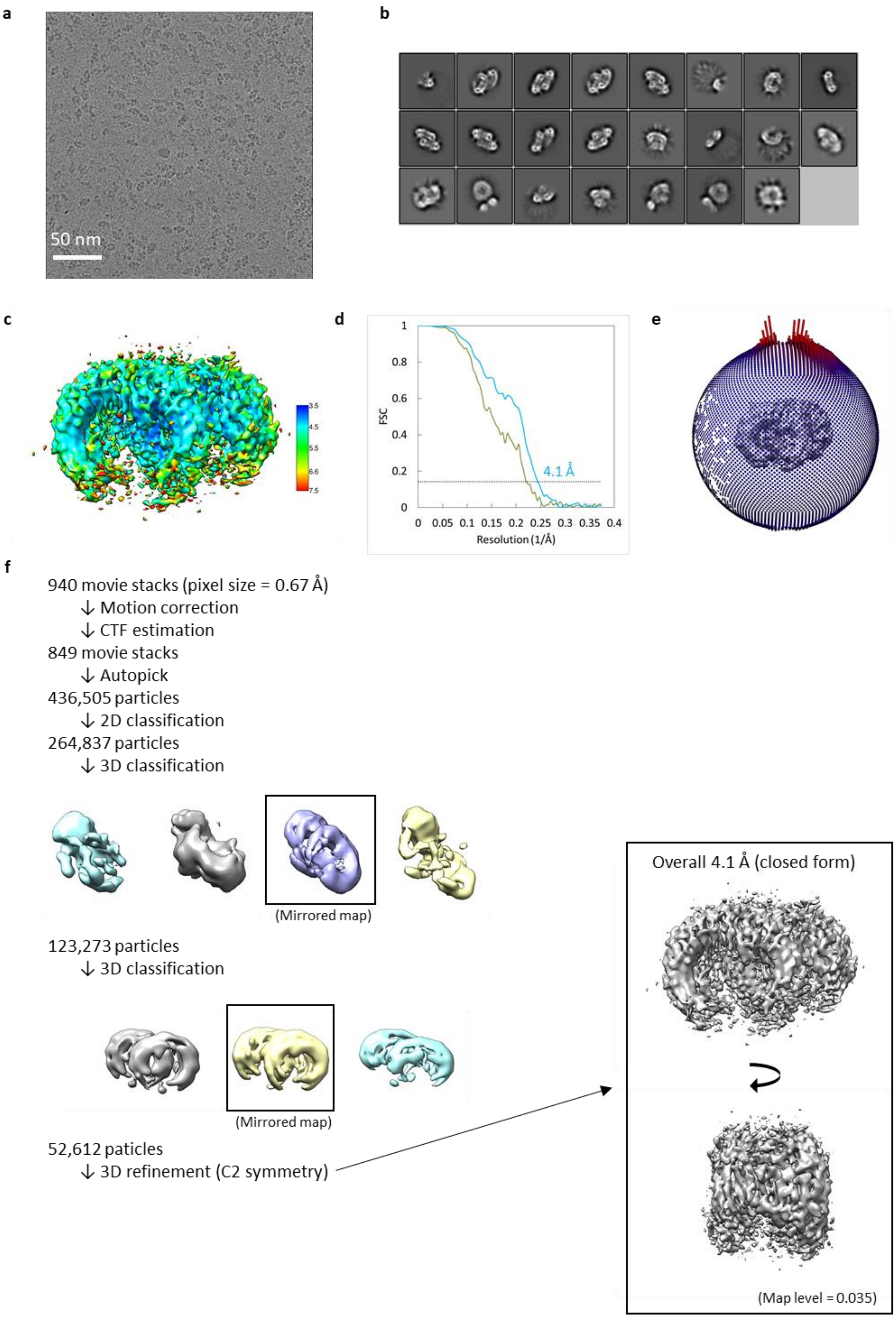
a
50 nm
b
c
3.5
4.5
5.5
6.6
7.5
d
FSC
4.1 Å
Resolution (1/Å)
e
f
940 movie stacks (pixel size = 0.67 Å)
↓ Motion correction
↓ CTF estimation
849 movie stacks
↓ Autopick
436,505 particles
↓ 2D classification
264,837 particles
↓ 3D classification
(Mirrored map)
123,273 particles
↓ 3D classification
(Mirrored map)
52,612 paticles
↓ 3D refinement (C2 symmetry)
Overall 4.1 Å (closed form)
(Map level = 0.035)

Supplementary Fig. 7. Cryo-EM analysis of TLR7/Cpd-3 complex

a) Representative micrograph with one experiment (white bar, 50 nm). **b)** 2D class averages selected for 3D classification. **c)** Cryo-EM density map of the final reconstruction of the TLR7/Cpd-3 complex, colored based on local resolution. **d)** Gold-standard FSC curve of the final 3D reconstruction (resolution cutoff at FSC = 0.143). Green and blue lines indicate the unmasked and the masked maps, respectively. **e)** Orientation distributions of the particles used for final reconstruction. **f)** Image-processing procedure.

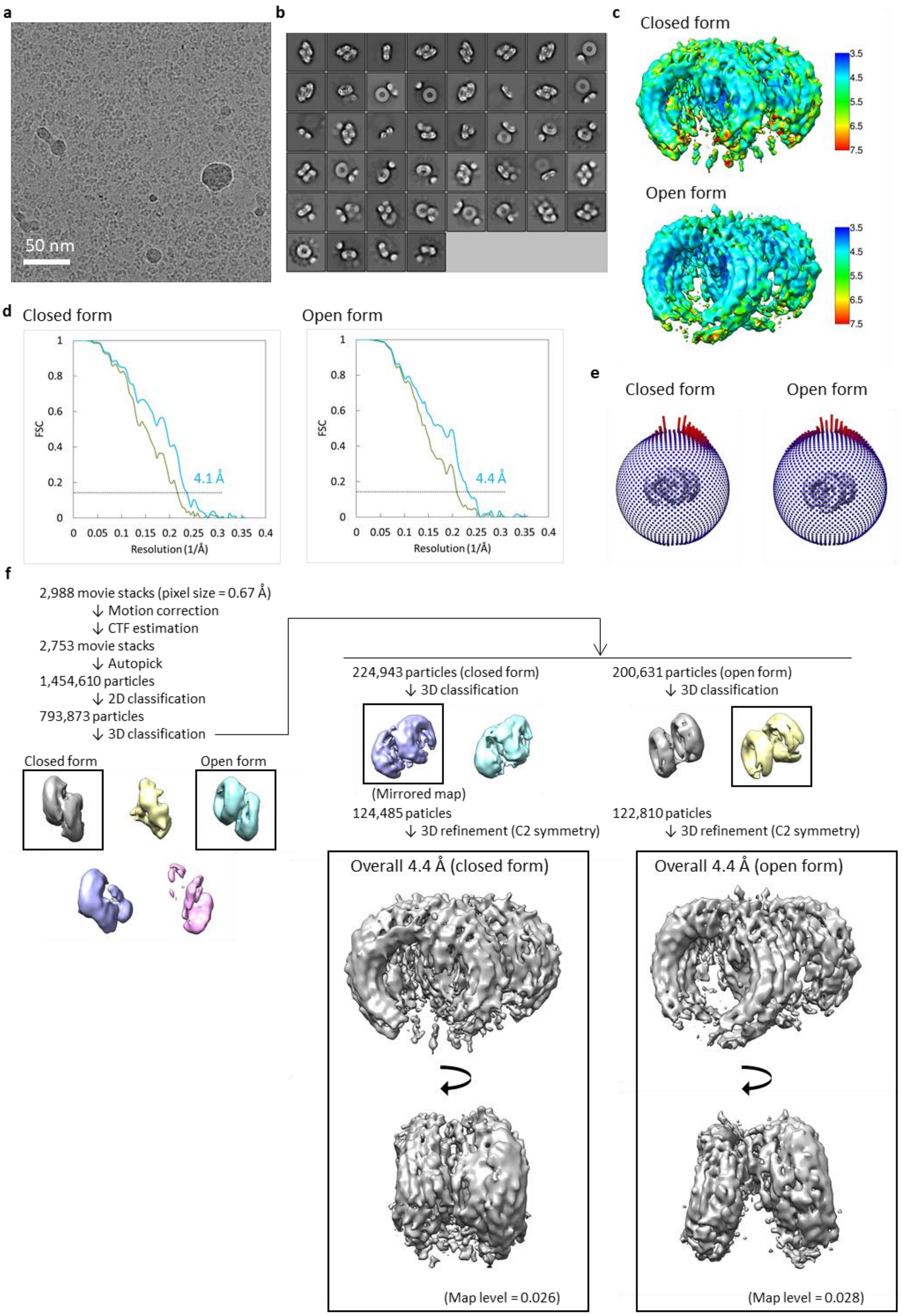
a
50 nm
b
c
Closed form
Open form
3.5
4.5
5.5
6.5
7.5
d
Closed form
Open form
FSC
4.1 Å
4.4 Å
Resolution (1/Å)
e
Closed form
Open form
f
2,988 movie stacks (pixel size = 0.67 Å)
↓ Motion correction
↓ CTF estimation
2,753 movie stacks
↓ Autopick
1,454,610 particles
↓ 2D classification
793,873 particles
↓ 3D classification
Closed form
Open form
224,943 particles (closed form)
↓ 3D classification
200,631 particles (open form)
↓ 3D classification
(Mirrored map)
124,485 paticles
↓ 3D refinement (C2 symmetry)
122,810 paticles
↓ 3D refinement (C2 symmetry)
Overall 4.4 Å (closed form)
Overall 4.4 Å (open form)
(Map level = 0.026)
(Map level = 0.028)

Supplementary Fig. 8. Cryo-EM analysis of TLR7/Cpd-6 complex

a) Representative micrograph with one experiment (white bar, 50 nm). **b)** 2D class averages selected for 3D classification. **c)** Cryo-EM density map of the final reconstructions of the closed (top) and open (bottom) forms of the TLR7/Cpd-6 complex, colored based on local resolution. **d)** Gold-standard FSC curves of the final 3D reconstructions (resolution cutoff at FSC = 0.143). Green and blue lines indicate the unmasked and the masked maps, respectively. **e)** Orientation distributions of the particles used for final reconstructions. **f)** Image-processing procedure.

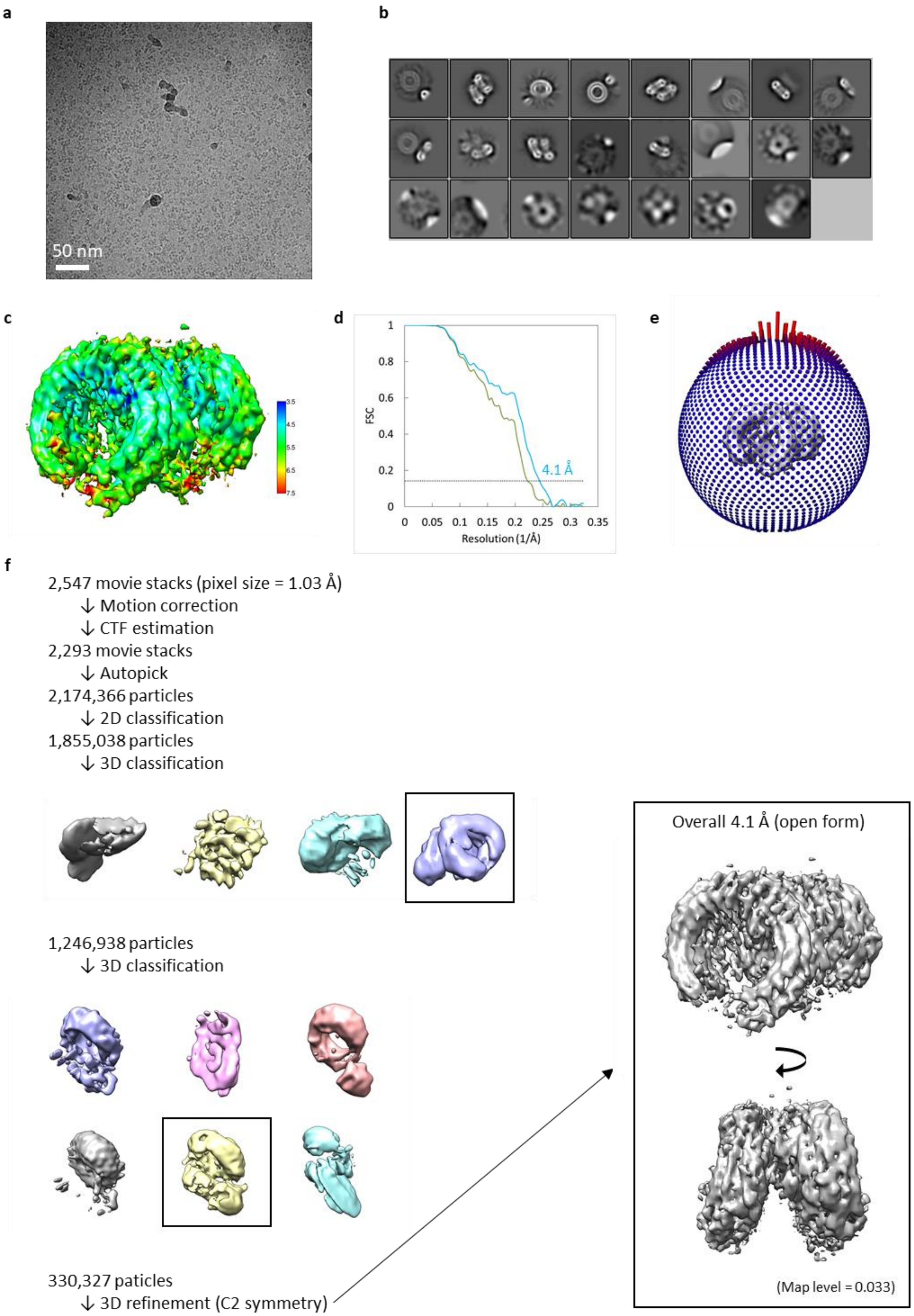
a
50 nm
b
c
3.5
4.5
5.5
6.5
7.5
d
1
0.8
0.6
0.4
0.2
0
FSC
4.1 Å
0 0.05 0.1 0.15 0.2 0.25 0.3 0.35
Resolution (1/Å)
e
f
2,547 movie stacks (pixel size = 1.03 Å)
↓ Motion correction
↓ CTF estimation
2,293 movie stacks
↓ Autopick
2,174,366 particles
↓ 2D classification
1,855,038 particles
↓ 3D classification
1,246,938 particles
↓ 3D classification
330,327 paticles
↓ 3D refinement (C2 symmetry)
Overall 4.1 Å (open form)
(Map level = 0.033)

Supplementary Fig. 9. Cryo-EM analysis of TLR7/Cpd-7 complex (Talos Arctica dataset)

a) Representative micrograph with one experiment (white bar, 50 nm). **b)** 2D class averages selected for 3D classification. **c)** Cryo-EM density map of the final reconstruction of the TLR7/Cpd-7 complex, colored based on local resolution. **d)** Gold-standard FSC curve of the final 3D reconstruction (resolution cutoff at FSC = 0.143). Green and blue lines indicate the unmasked and the masked map, respectively. **e)** Orientation distribution of the particles used for final reconstruction. **f)** Image-processing procedure.

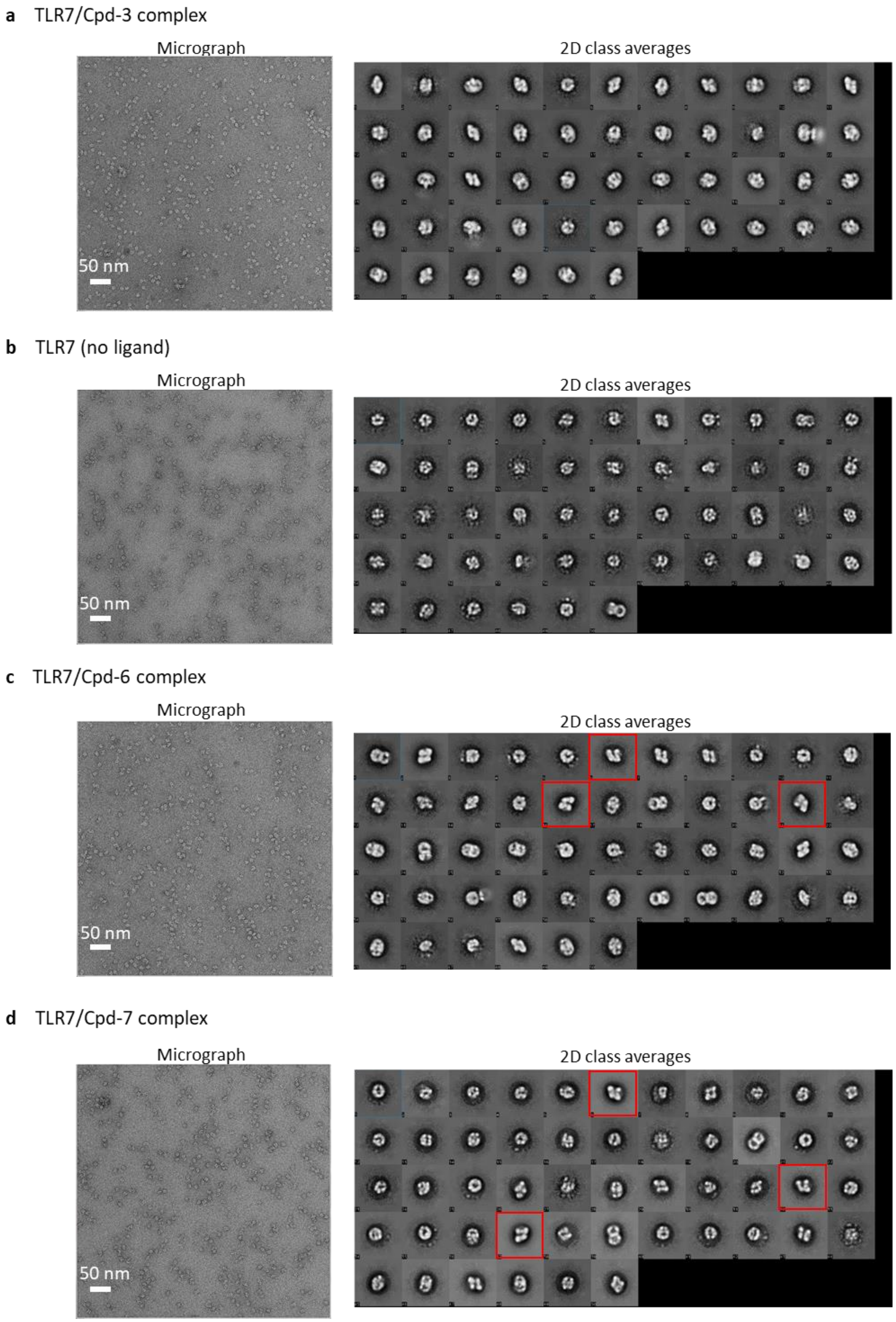
a TLR7/Cpd-3 complex
Micrograph
2D class averages
50 nm
b TLR7 (no ligand)
Micrograph
2D class averages
50 nm
c TLR7/Cpd-6 complex
Micrograph
2D class averages
50 nm
d TLR7/Cpd-7 complex
Micrograph
2D class averages
50 nm

Supplementary Fig. 10. Negative-staining EM analyses

a-d) Negative-staining EM analyses of crosslinked TLR7/Cpd-3, TLR7 (no ligand), TLR7/Cpd-6 and TLR7/CpD-7 complexes. Representative micrographs with one experiment (white bars, 50 nm) (left) and 2D class averages (right) are shown. The average images indicated by red squares are possible examples of the open form conformation of TLR7.

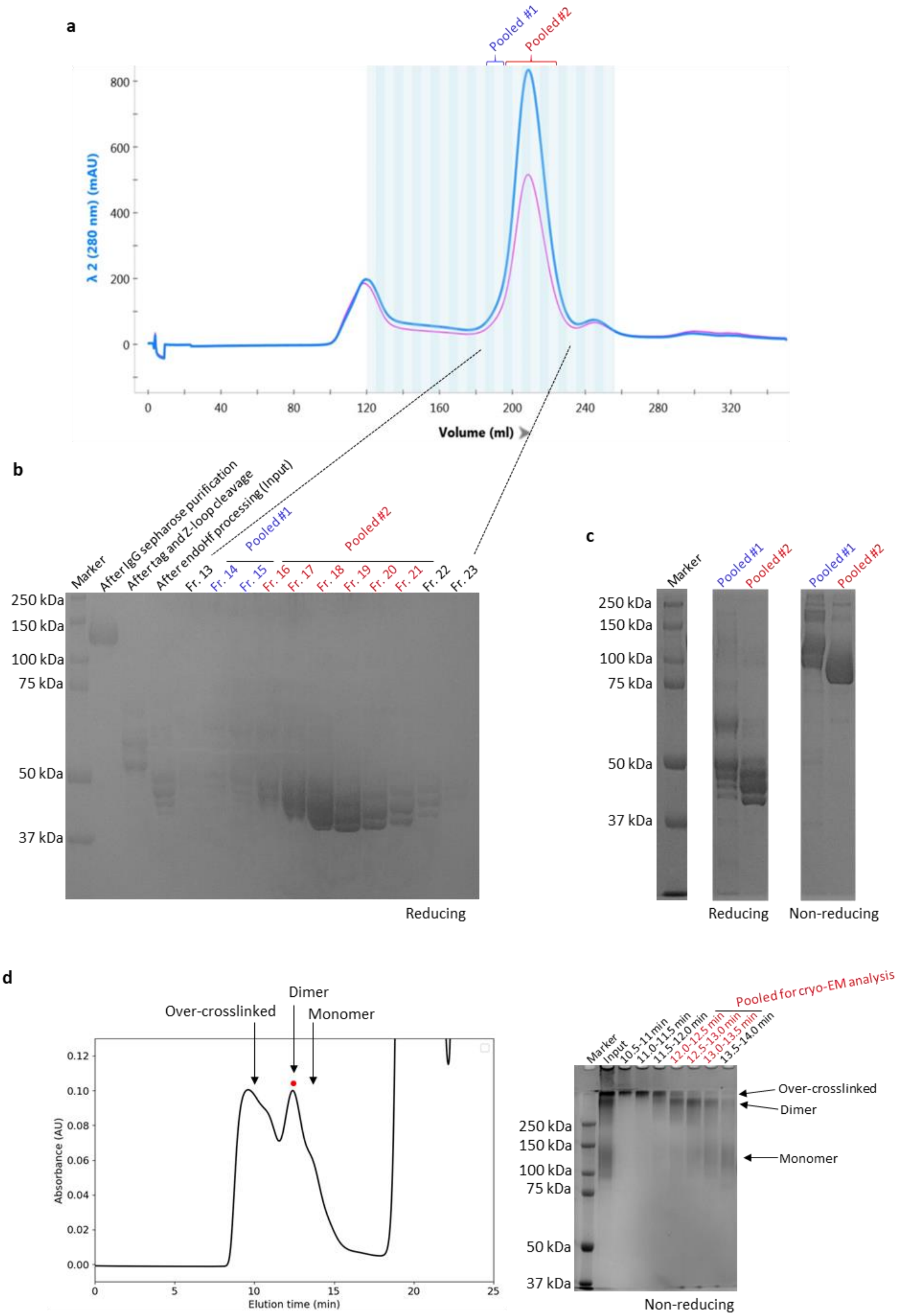

a
Pooled #1
Pooled #2
λ 2 (280 nm) (mAU)
800
600
400
200
0
0
40
80
120
160
200
240
280
320
Volume (ml)
b
Marker
After IgG sepharose purification
After tag and Z-loop cleavage
After endoHf processing (Input)
Fr. 13
Fr. 14
Fr. 15
Fr. 16
Fr. 17
Fr. 18
Fr. 19
Fr. 20
Fr. 21
Fr. 22
Fr. 23
Pooled #1
Pooled #2
250 kDa
150 kDa
100 kDa
75 kDa
50 kDa
37 kDa
Reducing
c
Marker
Pooled #1
Pooled #2
Pooled #1
Pooled #2
250 kDa
150 kDa
100 kDa
75 kDa
50 kDa
37 kDa
Reducing
Non-reducing
d
Over-crosslinked
Dimer
Monomer
Absorbance (AU)
0.12
0.10
0.08
0.06
0.04
0.02
0.00
0
5
10
15
20
25
Elution time (min)
Pooled for cryo-EM analysis
Marker
Input
10.5-11 min
11.0-11.5 min
11.5-12.0 min
12.0-12.5 min
12.5-13.0 min
13.0-13.5 min
13.5-14.0 min
Over-crosslinked
Dimer
Monomer
250 kDa
150 kDa
100 kDa
75 kDa
50 kDa
37 kDa
Non-reducing

Supplementary Fig. 11. Preparation of crosslinked TLR7/Cpd-7 complex with partially uncleaved protein A tag

a) Purification of glycan-trimmed TLR7 by gel filtration chromatography. Absorbances at 280 and 260 nm are indicated by blue and purple lines, respectively. **b)** SDS-PAGE analysis (reduced) of each stage before gel filtration purification and part of the eluates of the gel filtration chromatography are shown (bottom left). The SDS-page is a representative image with one experiment. **c)** SDS-PAGE analyses (reduced and non-reduced) of the pooled Fr. 14-15 and Fr.16-21 are also shown (bottom right). Fractions (Fr.) 14-15, which contained the partially uncleaved protein A tag, were used to prepare samples for cryo-EM analysis. Fr. 16-21 were used for crystallization. The SDS-page is a representative image with one experiment. **d)** Purification of crosslinked TLR7/Cpd-7 complex (left) by gel filtration chromatography after glutaraldehyde crosslinking. TLR7 proteins from Fr. 14-15 in **a** were used for this experiment. The red dot indicates the peak with subsequent pooling for further use. SDS-PAGE analysis (non-reducing) of the eluates of the gel filtration chromatography (right). Three fractions (red) were pooled for cryo-EM analysis using Titan Krios.

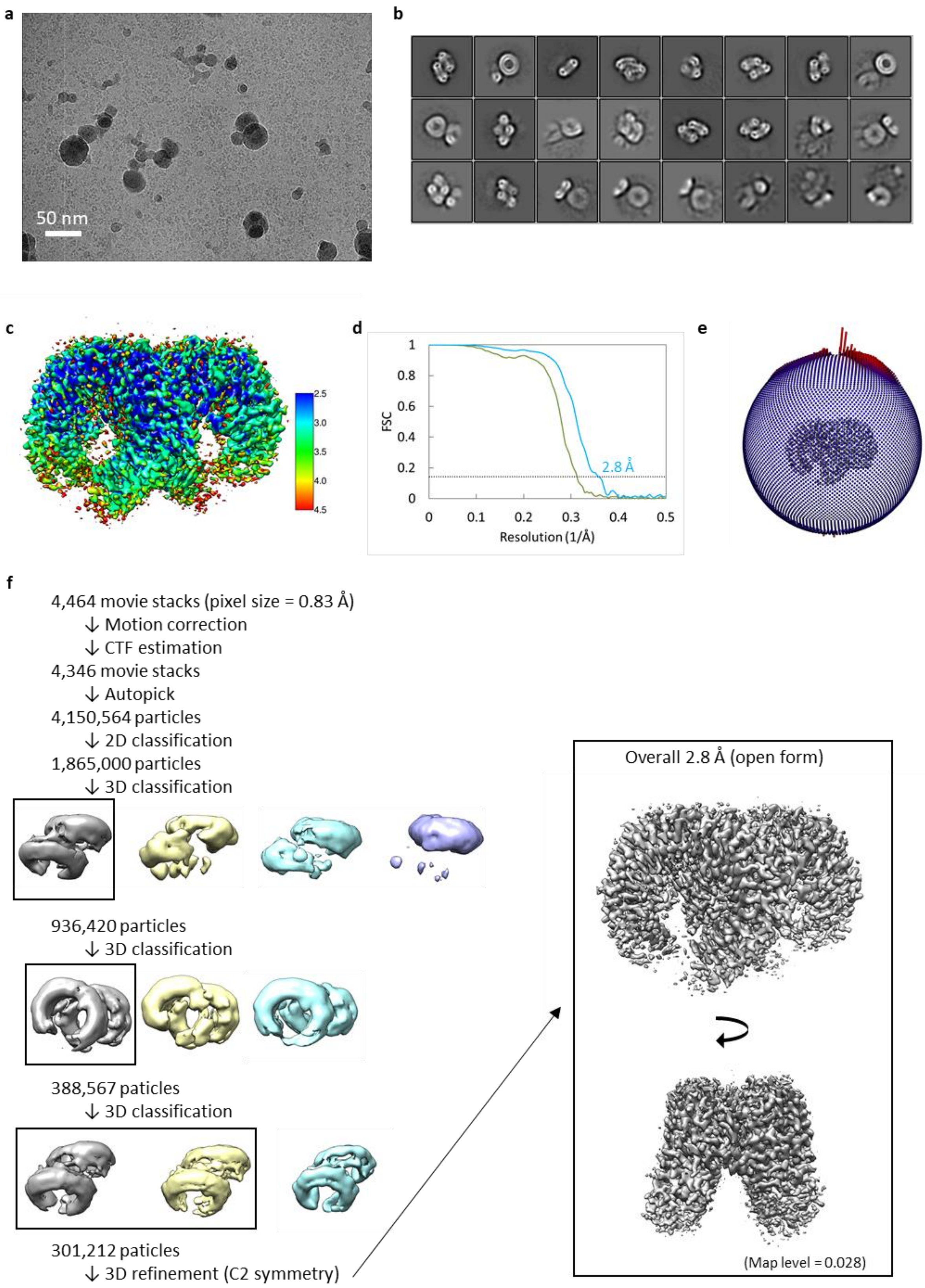
a
50 nm
b
c
2.5
3.0
3.5
4.0
4.5
d
FSC
2.8 Å
Resolution (1/Å)
e
f
4,464 movie stacks (pixel size = 0.83 Å)
↓ Motion correction
↓ CTF estimation
4,346 movie stacks
↓ Autopick
4,150,564 particles
↓ 2D classification
1,865,000 particles
↓ 3D classification
936,420 particles
↓ 3D classification
388,567 paticles
↓ 3D classification
301,212 paticles
↓ 3D refinement (C2 symmetry)
Overall 2.8 Å (open form)
(Map level = 0.028)

Supplementary Fig. 12. Cryo-EM analysis of TLR7/Cpd-7 complex (Titan Krios dataset)

a) Representative micrograph with one experiment (white bar, 50 nm). **b)** 2D class averages selected for 3D classification. **c)** Cryo-EM density map of the final reconstruction of the TLR7/Cpd-7 complex, colored based on local resolution. **d)** Gold-standard FSC curve of the final 3D reconstruction (resolution cutoff at FSC = 0.143). Green and blue lines indicate the unmasked and masked maps, respectively. **e)** Orientation distributions of the particles used for final reconstruction. **f)** Image-processing procedure.

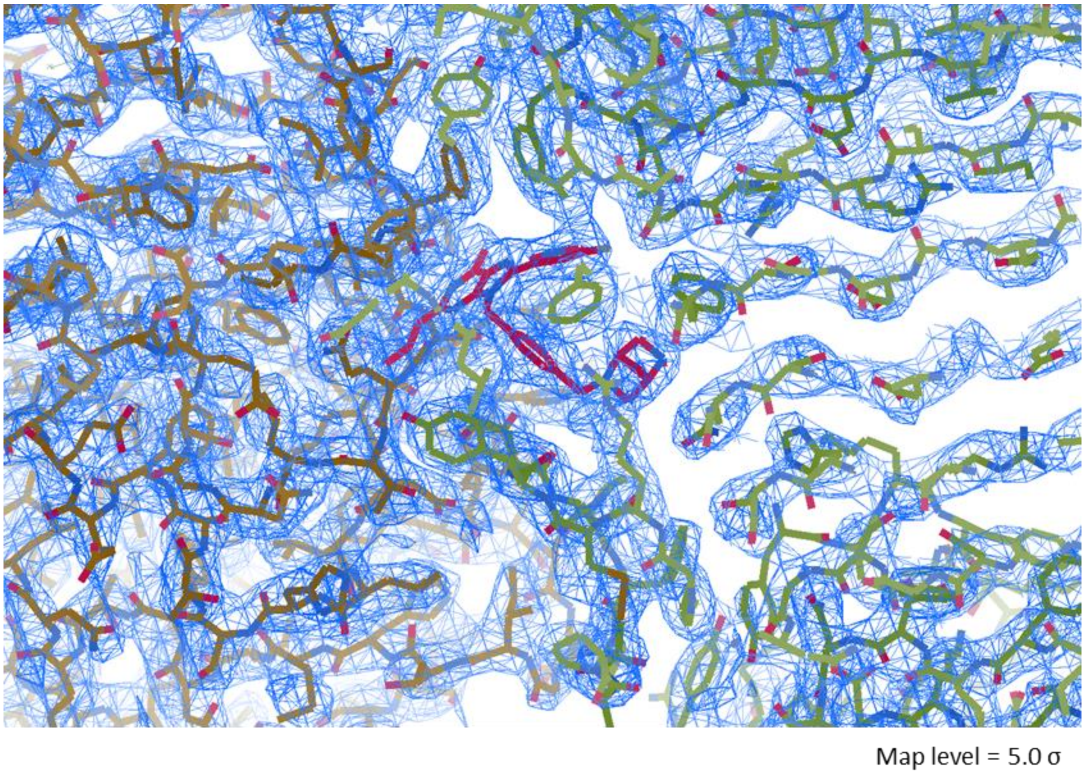

Supplementary Fig. 13. Cryo-EM densities around the Cpd-7 binding site

Cryo-EM densities of TLR7/Cpd-7 complex (Titan Krios dataset) is shown as blue meshes countered at 5.0 σ around the Cpd-7 binding site. The modeled structure is shown in stick representations. The C-atoms of two TLR7 protomers and Cpd-7 are colored brown, yellow and magenta, respectively.

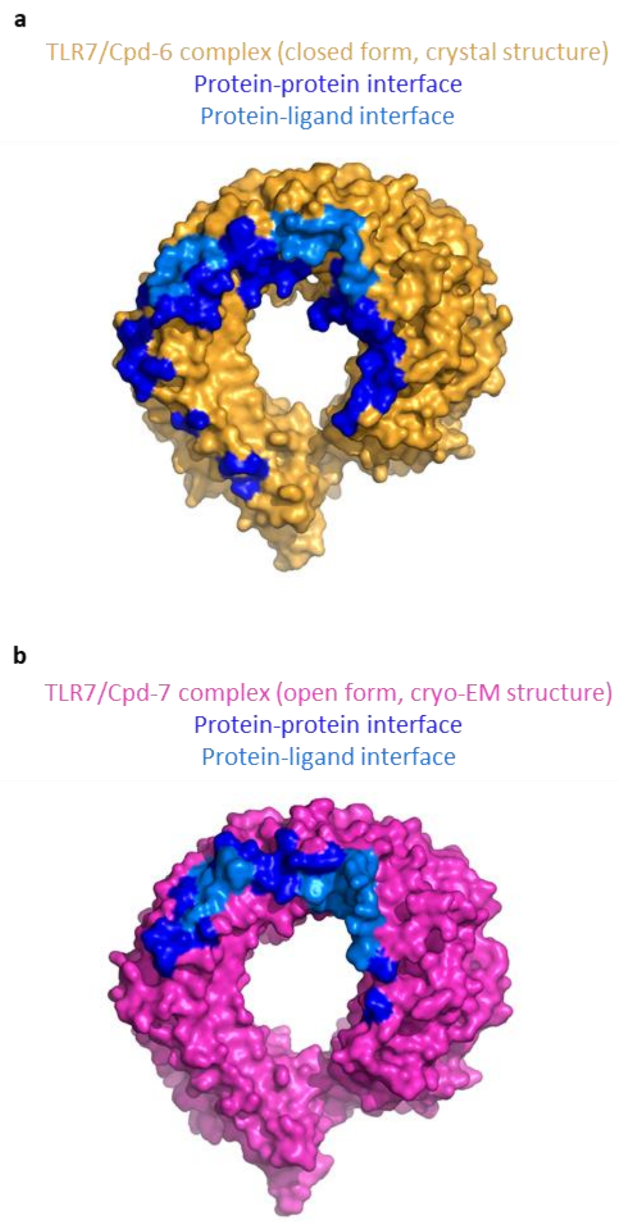

Supplementary Fig. 14. Comparison of TLR7 dimerization interfaces in the open and closed conformations

Protomers in the closed form of the TLR7 dimer (crystal structure of TLR7/Cpd-6 complex) in **a** and the open form of the TLR7 dimer (cryo-EM structure of TLR7/Cpd-7 complex) in **b** are shown in surface representation. The protein-protein interface and the protein-ligand interface are colored using dark and light blue, respectively.

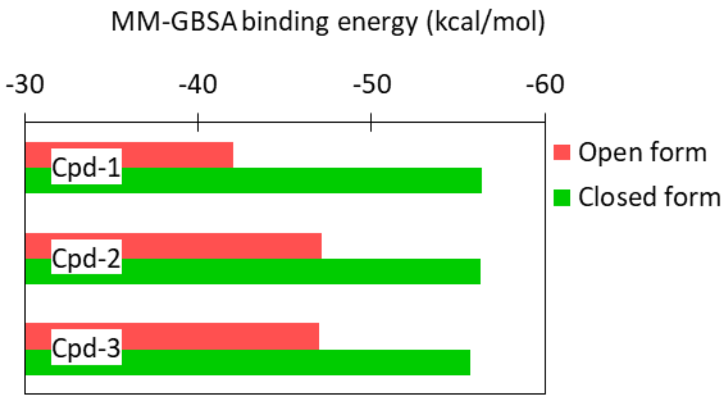

Supplementary Fig. 15. Calculation of the binding energy of agonists to TLR7

Molecular mechanics calculation of the binding energy of Cpd-1, Cpd-2 or Cpd-3 for both the open and closed forms.

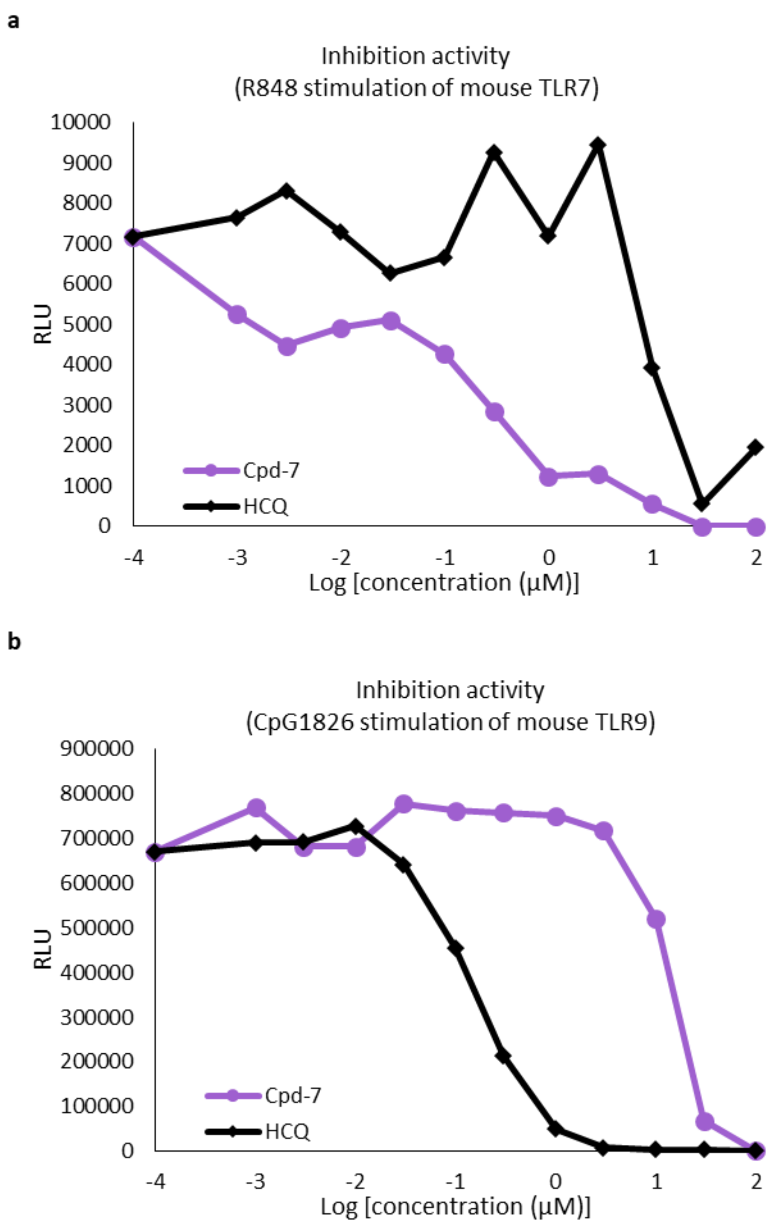

Supplementary Fig. 16. Selective inhibition of mouse TLR7 by Cpd-7

a) Dose-response curves for the inhibition of mouse TLR7 activity by Cpd-7 and hydroxychloroquine (positive control) measured by reporter gene assay with R848 (200 nM) as stimulating agent. Data are representative tests with three replicates. **b)** Dose-response curves for the inhibition of mouse TLR9 activity by Cpd-7 and hydroxychloroquine (positive control) measured by reporter gene assay with CpG1826 (100 nM) as stimulating agent. Data are representative tests with three replicates.

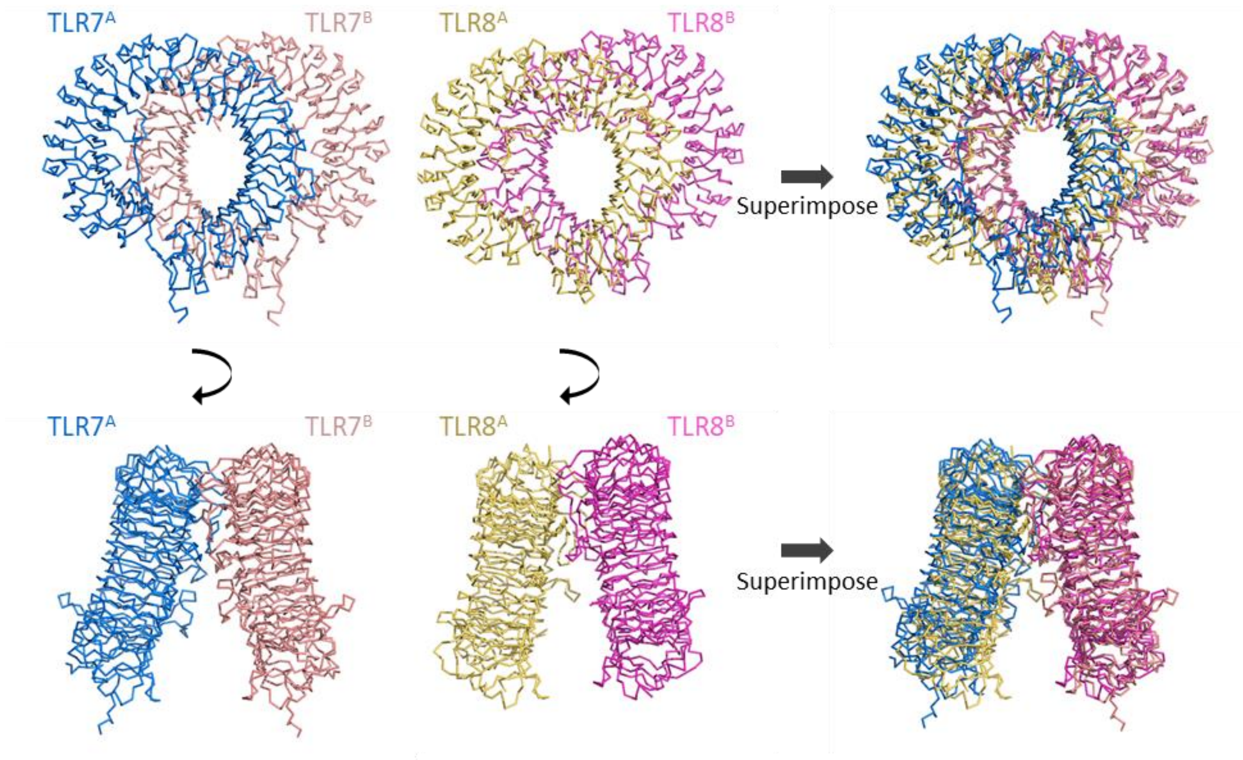

Supplementary Fig. 17. Comparison of TLR7/Cpd-7 complex with TLR8 inactive dimer

Front views and side views of TLR7/Cpd-7 complex (left) and TLR8 inactive dimer (PDB: 3W3G) (middle) are shown in ribbon representations. Two structures are superimposed in reference to their B protomers. The superimposed structures are shown on the right. $TLR7^A$ protomer is more apart from the B protomer compared to $TLR8^A$ protomer.

Supplementary Table 1. Crystallographic data collection and refinement statistic

**Data**	TLR7/Cpd-1 complex (closed form)	TLR7/Cpd-2 complex (closed form)	TLR7/Cpd-3 complex (closed form)	TLR7/Cpd-6 complex (closed form)
**PDB ID**	6LVX	6LVY	6LVZ	6LW0
**Data collection**				
Beamline	PF BL-5A	PF BL-5A	PF BL-5A	PF-AR NE3A
Wavelength (Å)	1.0000	1.0000	1.0000	1.0000
Software (reduction)	XDS	XDS	XDS	XDS
Space group	$P2_12_12_1$	$P2_12_12_1$	$P2_12_12_1$	$P2_12_12_1$
Cell dimensions				
a, b, c (Å)	98.8, 138.5, 148,5	99.0, 138.4, 148.7	98.6, 139.1, 149.2	98.2, 139.4, 149.0
Resolution (Å)	2.77 (2.86-2.77)	2.60 (2.66-2.60)	2.83 (2.92-2.83)	2.60 (2.66-2.60)
R_{meas}	0.110	0.142	0.116	0.119
$I/\sigma I$	27.4 (7.0)	20.8 (2.6)	16.2 (2.2)	13.8 (1.8)
Completeness (%)	100.0 (99.9)	100.0 (100.0)	100.0 (100.0)	100.0 (100.0)
Redundancy	13.0 (13.7)	13.4 (13.8)	6.7 (7.0)	6.7 (6.8)
**Refinement**				
Software (phasing)	molrep	molrep	molrep	molrep
Software (refinement)	Refmac5	Refmac5	Refmac5	Refmac5 Phenix.refine
Resolution (Å)	50.01-2.77	50.01-2.60	46.87-2.83	46.29-2.60
No. reflections	49,888	60,395	47,103	63,496
R_{work} / R_{free}	0.2120 / 0.2518	0.1944 / 0.2393	0.1871 / 0.2451	0.2054 / 0.2596
Model				
Protein	2 × TLR7	2 × TLR7	2 × TLR7	2 × TLR7
Ligand	2 × Cpd-1	2 × Cpd-2	2 × Cpd-3	2 × Cpd-6
Average B-factors (Å^2)	34.4	48.5	58.9	53.5
R.m.s deviations				
Bond lengths (Å)	0.013	0.013	0.010	0.010
Bond angles (º)	1.68	1.68	1.48	1.39

Each dataset was collected with one crystal. Highest resolution shell is shown in parenthesis

Supplementary Table 2. Cryo-EM data collection, processing and refinement statistics

**Data**	TLR7/Cpd-3 complex (closed form)	TLR7/Cpd-6 complex (closed form)	TLR7/Cpd-6 complex (open form)	TLR7/Cpd-7 complex (open form)	TLR7/Cpd-7 complex (open form)
**EMDB ID**	EMD-0999	EMD-1000	EMD-30000	EMD-30001	EMD-30002
**PDB ID**	-	-	-	-	6LW1
**Data collection and processing**					
**Cryo-EM facility**	RIKEN RSC Cryo-EM facility			University of Tokyo	
Microscope	Glacios	Glacios		Talos Arctica	Titan Krios
Voltage (kV)	200	200		200	300
Detector	Falcon 3EC	Falcon 3EC		K2 summit	K3
Magnification	150,000	150,000		130,000	105,000
Pixel size (Å)	0.67	0.67		1.03	0.83
Total dose ($e^-/Å^2$)	50	50		50	60
Total movie stacks	940	2,988		2,547	4,464
Initial particle images (no.)	436,505	1,454,610		2,174,366	4,150,564
Final particle images (no.)	52,612	224,943	200,631	330,327	301,212
Symmetry imposed	C2	C2	C2	C2	C2
Map resolution (Å)	4.1	4.4	4.4	4.1	2.8
FSC threshold	0.143	0.143	0.143	0.143	0.143
Map sharpening B factor (Å2)	-93.4	-202.2	-214.0	-195.3	-69.8
**Refinement**					
Initial model used (PDB ID)	-	-	-	-	5GMH
Software	-	-	-	-	Chimera, COOT, Phenix
Model resolution (Å)	-	-	-	-	3.0
FSC threshold	-	-	-	-	0.5
Model composition	-	-	-	-	
Protein	-	-	-	-	TLR7 × 2
Ligand	-	-	-	-	Cpd-7 × 2
Average B-factors ($Å^2$)	-	-	-	-	
Protein	-	-	-	-	72.3
Ligand	-	-	-	-	17.1
R.m.s deviations	-	-	-	-	
Bond lengths (Å)	-	-	-	-	0.009
Bond angles (º)	-	-	-	-	0.96
Validation	-	-	-	-	
Molprobity score	-	-	-	-	2.79
Clashscore	-	-	-	-	11.25
Poor rotamers (%)	-	-	-	-	7.1
Ramachandran plot	-	-	-	-	
Favored (%)	-	-	-	-	89.2
Allowed (%)	-	-	-	-	10.3
Outliers (%)	-	-	-	-	0.5

Synthetic procedures and characterization

General information

Melting points were determined on a Stanford Research Systems *Opti Meit*. Specific rotations were recorded on JASCO *P-1020* polarimeter using $CHCl_3$ as a solvent. NMR spectra were recorded on a JEOL *JNM-AL400* spectrometer. Splitting patterns were designated as "s, d, t, q, m, and br" indicating "singlet, doublet, triplet, quartet, multiplet, and broad", respectively. Chemical shifts (δ) are reported with DMSO (δ = 2.49 ppm) or $CHCl_3$ (δ = 7.24 ppm) as internal standard. All *J* values are given in Hz. High-resolution mass spectra (HRMS) were recorded on a Thermo Fisher Scientific *Orbitrap Elite* MS equipment. Reagents and solvents were used as obtained from commercial suppliers without further purification. Column chromatography was carried out using a Yamazen *W-prep* system, and performed using pre-packed silica-gel (SiO_2) or amino silica-gel (amino SiO_2) columns. Reaction progress was monitored by TLC analysis on a silica-gel or amino silica-gel coated glass plate and liquid chromatography-mass spectrometry (LC/MS). Visualization was done with UV light (254 nm) or iodine. LC/MS was performed using a Waters ACQUITY UPLC *TM* system. The sample was dissolved in acetonitrile or methanol solution, applied on a Waters ACQUITY UPLC *TM* BEH C18 column (2.1 mm × 30 mm, 1.7 µm), eluted at 0.8 mL/min with a 0.0-1.3 min gradient (from 2% B to 96% B), where solvent A is water (0.06% formic acid solution) and solvent B is acetonitrile (0.06% formic acid solution) at 40 °C, and detected by Waters ACQUITY *TM* SQ detector with UV light (220 nm and 254 nm). All reactions were carried out under a nitrogen atmosphere unless otherwise mentioned. HPLC was performed using a SHIMADZU *Prominence UFLC*™ system. The sample was dissolved in acetonitrile or methanol solution, applied on a Phenomenex® Kinetex® C18 column (3.0 mm × 75 mm, 2.6 µm), and eluted at 0.9 mL/min with a 8 min gradient (from 1% B to 99% B), where solvent A is water (0.05% TFA solution) and solvent B is acetonitrile (0.05% TFA solution) at 40 °C. The purities were determined by HPLC.

6-Amino-9-benzyl-2-butoxy-7,9-dihydro-8*H*-purin-8-one (Cpd-1: SM-374527)

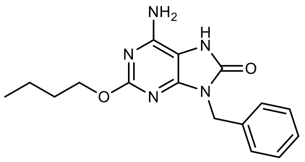

Cpd-1 was prepared according to the literature as white solids [1]. 287-289 °C (decomposition) (lit.1, >300 °C); ^{1}H-NMR (400 MHz, DMSO-d_6): δ 9.93 p.p.m. (s, 1H), 7.33-7.22 (m, 5H), 6.42 (s, 2H), 4.84 (s, 2H), 4.13 (t, J = 6.6 Hz, 2H), 1.64-1.57 (m, 2H), 1.40-1.31 (m, 2H), 0.89 (t, J = 7.6, 3H); ^{13}C-NMR (100 MHz, DMSO-d_6): δ 160.1 p.p.m., 152.2, 149.2, 147.7, 137.2, 128.4 (2C), 127.4 (2C), 127.3, 98.2, 70.2, 65.8, 42.3, 30.5, 18.7, 13.7; HRMS (*m/z*): $[M+H]^+$ calcd. for $C_{16}H_{20}N_5O_2$, 314.1612; found, 314.1616; HPLC purity = 99.8% (Rt = 3.478 min).

6-Amino-9-benzyl-2-(2-methoxyethoxy)-7,9-dihydro-8*H*-purin-8-one (Cpd-2: SM-360320)

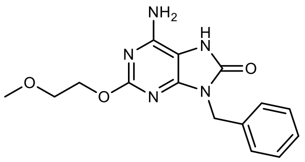

Cpd-2 was prepared according to the literature as white solids [2]. 281-283 °C (decomposition) (lit.2, 290-292 °C); ^{1}H-NMR (400 MHz, DMSO-d_6): δ 9.96 p.p.m. (s, 1H), 7.33-7.22 (m, 5H), 6.45 (s, 2H), 4.84 (s, 2H), 4.25 (t, J = 4.8 Hz, 2H), 3.57 (t, J = 4.8, 2H), 3.25 (s, 3H); ^{13}C-NMR (100 MHz, DMSO-d_6): δ 159.8 p.p.m., 152.2, 149.2, 147.7, 137.1, 128.5 (2C), 127.4 (2C), 127.3, 98.3, 70.2, 65.2, 58.0; HRMS (*m/z*): $[M+H]^+$ calcd. for $C_{15}H_{18}N_5O_3$, 316.1404; found, 316.1408; HPLC purity = 99.8% (Rt = 3.15 min).

6-Amino-2-(2-methoxyethoxy)-9-(3-pyridyl)-7,9-dihydro-8*H*-purin-8-one (Cpd-3: SM-394830)

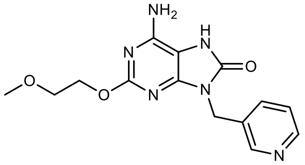

Cpd-3 was prepared according to the literature as white solids [3]. m.p.: 255-258 °C (decomposition); ^{1}H-NMR (400 MHz, DMSO-d_6): δ 9.98 p.p.m. (s, 1H), 8.57-8.54 (m, 1H), 8.47 (dd, J = 2.4, 4.8 Hz), 7.71-7.66 (m,

1H), 7.37-7.32 (m, 1H), 6.47 (s, 2H), 4.89 (s, 2H), 4.25 (t, *J* = 4.8 Hz, 2H), 3.57 (t, *J* = 4.8, 2H), 3.26 (s, 3H); ^{13}C-NMR (100 MHz, DMSO-d_6): δ 159.8 p.p.m., 152.1, 149.0, 148.7, 147.8, 135.4 (2C), 132.7, 123.6, 98.4, 70.2, 65.3, 58.0, 40.1; HRMS (*m/z*): $[M+H]^+$ calcd. for $C_{14}H_{17}N_6O_3$, 317.1357; found, 317.1354; HPLC purity = 98.9% (Rt = 2.11 min).

9-Benzyl-2-butoxy-8-(pyridin-3-yl)-9*H*-purin-6-amine (Cpd 5: DSR-070543)

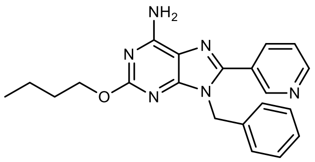

To a suspension of methyl 9-benzyl-8-bromo-2-butoxy-9*H*-purin-6-amine[4] (120 mg, 0.319 mmol) in 1,4-dioxane (3 ml) and water (0.5 ml) were added pyridine-3-boronic acid (43.2 mg, 0.351 mmol), tetrakis(triphenylphosphine)palladium (37.0 mg, 0.032 mmol), and potassium carbonate (90.1 mg, 0.652 mmol) at room temperature. After stirring at reflux temperature for 4 h, the reaction mixture was diluted with satd. $NaHCO_3$ aq. (20 ml) and extracted with $CHCl_3$ (30 ml x 2). The combined organic layer was dried over Na_2SO_4, filterted, and concentrated. The residue was purified by flash column chromatography (SiO_2, CHCl3-MeOH) to get 9-benzyl-2-butoxy-8-(pyridin-3-yl)-9*H*-purin-6-amine (**Cpd 5**) (58.2 mg, 48.7 % yield) as white solids. m.p.: 177-178 °C; ^{1}H-NMR (400 MHz, DMSO-d_6): δ 8.81 p.p.m. (d, *J* = 1.8 Hz, 1H), 8.63 (dd, *J* = 1.2, 4.9 Hz, 1H), 8.02 (dt, *J* = 1.8, 7.9 Hz, 1H), 7.48 (dd, *J* = 4.9, 7.9 Hz, 1H), 7.42 (brs, 2H), 7.27-7.19 (m, 3H), 6.97 (d, *J* = 6.7 Hz, 2H), 5.43 (s, 2H), 4.21 (t, *J* = 6.7 Hz, 2H), 1.64 (tt, *J* = 6.7, 7.9 Hz, 2H), 1.38 (qt, *J* = 7.3, 7.9 Hz, 2H), 0.90 (t, *J* = 7.3 Hz, 3H); ^{13}C-NMR (100 MHz, DMSO-d_6): δ 192.6 p.p.m., 161.8, 159.8, 157.2, 157.0, 153.3, 145.0, 144.0, 143.2, 138.6, 138.4, 135.4, 129.9, 127.6, 127.1, 122.7, 122.5, 115.3, 62.1, 45.7, 14.5; HRMS (*m/z*): $[M+H]^+$ calcd. for $C_{21}H_{23}N_6O$, 375.1928; found, 375.1924; HPLC purity = 99.9% (Rt = 3.72 min).

Methyl 4-[(6-amino-2-ethoxy-9*H*-purin-9-yl)methyl]benzoate

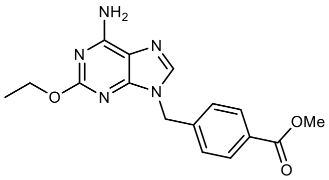

To a suspension of 2-Ethoxy-9H-purin-6-amine [5] (3.45 g, 19.3 mmol) and 4-(bromomethyl)benzoate (5.30 g, 23.1 mmol) in DMF (35 ml) was added potassium carbonate (6.65 g, 48.1 mmol) at 0 ºC. After stirring at room temperature for 3.5 h, the reaction mixutre was diluted with satd. $NaHCO_3$ aq. (70 ml) and water (35 ml) at 0 °C, and stirred at the same temperature for 1 h. The precipitates were filtered and washed with water (10 ml x 4). The obtained solids were suspended in MeCN (50 ml) and stirred at 80 °C for 1 h, at room temperature for 2 h, and at 0 °C for 1 h. The precipitates were collected and washed with with MeCN (15 ml x 3) to afford methyl 4-[(6-amino-2-ethoxy-9H-purin-9-yl)methyl]benzoate (4.11 g, 12.6 mmol, 65% yield) as white solids. m.p.: 202-204 °C; ^{1}H-NMR (400 MHz, DMSO-*d*$_6$): δ 8.05 p.p.m. (s, 1H), 7.92 (d, *J* = 7.9 Hz, 2H), 7.40 (d, *J* = 7.9 Hz, 2H), 7.21 (brs, 2H), 5.34 (s, 2H), 4.21 (q, *J* = 7.3 Hz, 2H), 3.82 (s, 3H), 1.24 (t, *J* = 7.3 Hz, 3H); ^{13}C-NMR (100 MHz, DMSO-*d*$_6$): δ 165.8 p.p.m., 161.4, 156.8, 151.2, 142.5, 139.4, 129.5 (2C), 128.9, 127.7 (2C), 115.0, 61.8, 52.1, 45.6, 14.5; HRMS (*m/z*): $[M+H]^+$ calcd. for $C_{16}H_{18}N_5O_3$, 328.1404; found, 328.1399.

Methyl 4-[(6-amino-8-bromo-2-ethoxy-9*H*-purin-9-yl)methyl]benzoate

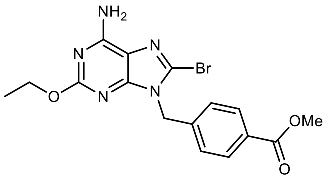

To a suspension of methyl 4-[(6-amino-2-ethoxy-9H-purin-9-yl)methyl]benzoate (23.7 g, 72.4 mmol) and sodium acetate (11.89 g, 145 mmol) in MeOH (84 ml) and $CHCl_3$ (420 ml) was added bromine (5.43 ml, 109 mmol) in $CHCl_3$ (15 ml) over 15 min at 4-6 ºC. After stirring at the same temperature for 2 h, the reaction mixutre was quenched by satd. $NaHCO_3$ aq. (200 ml) and satd. $Na_2S_2O_3$ aq. (200 ml). After stirring at 0 ºC for 10 min, the mixture was extracted with $CHCl_3$ (400 ml x 2). The combined organic layer was

dried over Na_2SO_4, filtered, and concentrated. The residue was suspended in MeCN (260 ml) and stirred at 80 °C for 45 min. Then, the mixture was cooled down to room temperature over 30 min and stirred at 0 °C for 1 h. The precipitates were filtered, washed with MeCN (50 ml x 4), and dried in vacuo to afford methyl 4-[(6-amino-8-bromo-2-ethoxy-9*H*-purin-9-yl)methyl]benzoate (20.92 g, 51.5 mmol, 71.1% yield) as white solids. m.p.: 218-220 °C; ^{1}H-NMR (400 MHz, DMSO-d_6): δ 7.94 p.p.m. (d, J = 7.9 Hz, 2H), 7.44 (brs, 2H), 7.33 (d, J = 7.9 Hz, 2H), 5.33 (s, 2H), 4.22 (q, J = 7.3 Hz, 2H), 3.82 (s, 3H), 1.25 (t, J = 7.3 Hz, 3H); ^{13}C-NMR (100 MHz, $CDCl_3$): δ 166.5 p.p.m., 162.0, 155.1, 153.1, 140.1, 130.1 (2C), 127.7 (2C), 124.5, 116.1, 63.3, 52.2, 46.9, 14.5; HRMS (*m/z*): $[M+H]^+$ calcd. for $C_{16}H_{17}BrN_5O_3$, 406.0509; found, 406.0508.

Methyl 4-{[6-amino-2-ethoxy-8-(5-fluoropyridin-3-yl)-9*H*-purin-9-yl]methyl}benzoate

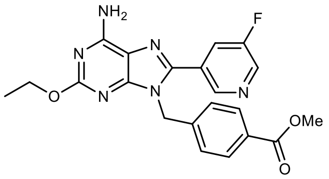

To a suspension of methyl 4-[(6-amino-8-bromo-2-ethoxy-9*H*-purin-9-yl)methyl]benzoate (31.6 g, 78 mmol) in 1,4-dioxane (320 ml) and water (80 ml) were added 3-fluoropyridine-5-boronic acid pinacol ester (19.1 g, 86 mmol), tetrakis(triphenylphosphine)paradium (9.00 g, 7.79 mmol), and potassium carbonate (21.5 g, 156 mmol) at room temperature. After stirring at reflux temperature for 5 h, the reaction mixture was diluted with satd. $NaHCO_3$ aq. (420 ml) at room temperature and extracted with $CHCl_3$-MeOH (20:1) (700 ml x 1, 500 ml x1). The combined organic layer was dried over Na_2SO_4 and concentrated. The residue was suspended in MeCN (500 ml) and stirred at 70 °C for 1 h. Then, the mixture was cooled down to room temperature over 2 h and stirred at 0 °C for 30 min. The precipitates were filtered, washed with MeCN (100 ml x 3), and dried in vacuo to afford methyl 4-{[6-amino-2-ethoxy-8-(5-fluoropyridin-3-yl)-9*H*-purin-9-yl]methyl}benzoate (27.7 g, 65.6 mmol, 84% yield) as white solids. m.p.: 183-185 °C; ^{1}H-NMR (400 MHz, DMSO-d_6): δ 8.66-8.64 p.p.m. (m, 2H), 7.98-7.94 (m, 1H), 7.84 (d, J = 7.9 Hz, 2H), 7.51 (brs, 2H), 7.12

(d, J = 7.9 Hz, 2H), 5.55 (s, 2H), 4.25 (q, J = 7.3 Hz, 2H), 3.81 (s, 3H), 1.25 (t, J = 7.3 Hz, 3H); ^{13}C-NMR (100 MHz, $CDCl_3$): δ 166.4 p.p.m., 162.5, 159.0 (d, $J_{(C\text{-}F)}$ = 259.1 Hz), 156.42, 154.0, 145.3 (d, $J_{(C\text{-}F)}$ = 1.9 Hz), 144.86 (d, $J_{(C\text{-}F)}$ = 4.8 Hz), 140.9, 139.3 (d, $J_{(C\text{-}F)}$ = 23.1 Hz), 130.4 (2C), 130.0, 127.47 (d, $J_{(C\text{-}F)}$ = 3.9 Hz), 126.6 (2C), 122.8 (d, $J_{(C\text{-}F)}$ = 20.2 Hz), 115.9, 63.4, 52.2, 46.7, 14.5; HRMS (*m/z*): $[M+H]^+$ calcd. for $C_{21}H_{20}FN_6O_3$, 423.1575; found, 423.157.

(4-{[6-Amino-2-ethoxy-8-(5-fluoropyridin-3-yl)-9*H*-purin-9-yl]methyl}phenyl)methanol

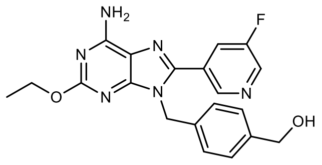

To a suspension of methyl 4-{[6-amino-2-ethoxy-8-(5-fluoropyridin-3-yl)-9*H*-purin-9-yl]methyl}benzoate (550 mg, 1.30 mmol) in THF (75 ml) was added diisobutylaluminum hydride (1.0 M toluene solution; 5.6 ml, 5.60 mmol) over 3 min at 0 ºC. After stirring at the same temperature for 1.5 h, diisobutylaluminum hydride (1.0 M toluene solution; 2.6 ml, 2.60 mmol) was added over 2 min at 0 ºC. After stirring at the same temperature for 20 min, the reaction mixture was diluted with AcOEt (5 ml) and quenched by satd. Rochelle salts aq. (50 ml) at 0 ºC. After stirring at room temperature for 2 h, the mixture was extracted with $CHCl_3$ (100 ml x2). The combined organic layer was dried over Na_2SO_4, filtered, and concentrated. The residue was purified by flash column chromatography (SiO_2, $CHCl_3$-MeOH) to give (4-{[6-amino-2-ethoxy-8-(5-fluoropyridin-3-yl)-9*H*-purin-9-yl]methyl}phenyl)methanol (416 mg, 81% yield) as white solids. m.p.: 188-190 °C; ^{1}H-NMR (400 MHz, DMSO-d_6): δ 8.70 p.p.m. (dd, J = 1.2, 1.8 Hz, 1H), 8.66 (d, J = 3.1 Hz, 1H), 8.01-7.96 (m, 1H), 7.46 (brs, 2H), 7.19 (d, J = 7.9 Hz, 2H), 6.94 (d, J = 7.9 Hz, 2H), 5.45 (s, 2H), 5.12 (t, J = 5.5 Hz, 1H), 4.40 (d, J = 5.5 Hz, 2H), 4.26 (q, J = 6.7 Hz, 2H), 1.27 (t, J = 6.7 Hz, 3H); ^{13}C-NMR (100 MHz, DMSO-d_6): δ 161.8 p.p.m., 158.5 (d, $J_{(C\text{-}F)}$ = 255.3 Hz), 156.9, 153.3, 145.0 (d, $J_{(C\text{-}F)}$ = 3.9 Hz), 144.1 (d, $J_{(C\text{-}F)}$ = 1.9 Hz), 141.9, 138.4 (d, $J_{(C\text{-}F)}$ = 23.1 Hz), 134.9, 127.8 (d, $J_{(C\text{-}F)}$ = 3.9 Hz), 126.8 (2C), 126.1 (2C), 122.5 (d, $J_{(C\text{-}F)}$ = 20.2 Hz), 115.3, 62.4, 62.0, 45.6, 14.5; HRMS (*m/z*): $[M+H]^+$ calcd. for $C_{20}H_{20}FN_6O_2$, 395.1626; found, 395.1623.

4-{[6-Amino-2-ethoxy-8-(5-fluoropyridin-3-yl)-9*H*-purin-9-yl]methyl}benzaldehyde

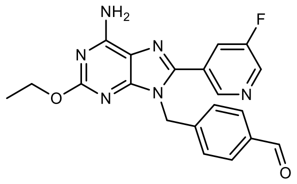

To a solution of (4-{[6-amino-2-ethoxy-8-(5-fluoropyridin-3-yl)-9*H*-purin-9-yl]methyl}phenyl)methanol (866 mg, 2.20 mmol) in THF (50 ml) was added MnO_2 (1.50 g, 14.7 mmol) at room temperature. After stirring at the same temperature for 2 days, the reaction mixture was filtered through a celite pad and the pad was washed with THF (100 ml). The filtrate was concentrated. The residue was purified by flash column chromatography (SiO_2, $CHCl_3$-MeOH) to 4-{[6-amino-2-ethoxy-8-(5-fluoropyridin-3-yl)-9*H*-purin-9-yl]methyl}benzaldehyde (667 mg, 77% yield) as white solids. m.p.: 185-187 °C; ^{1}H-NMR (400 MHz, DMSO-d_6): δ 9.92 p.p.m. (s, 1H), 8.68 (dd, J = 1.2, 1.8 Hz, 1H), 8.65 (d, J = 3.2 Hz, 1H), 8.00-7.95 (m, 1H), 7.82-7.78 (m, 2H), 7.50 (brs, 2H), 7.21 (d, J = 8.3 Hz, 2H), 5.57 (s, 2H), 4.25 (q, J = 7.0 Hz, 2H), 1.25 (t, J = 7.0 Hz, 3H); ^{13}C-NMR (100 MHz, DMSO-d_6): δ 192.6 p.p.m., 161.8, 158.5 (d, $J_{(C\text{-}F)}$ = 255.3 Hz), 157.0, 153.3, 145.1 (d, $J_{(C\text{-}F)}$ = 3.9 Hz), 144.0 (d, $J_{(C\text{-}F)}$ = 1.9 Hz), 143.2, 138.5 (d, $J_{(C\text{-}F)}$ = 23.1 Hz), 135.4, 129.9 (2C), 127.6 (d, $J_{(C\text{-}F)}$ = 4.8 Hz),, 127.1 (2C), 122.6 (d, $J_{(C\text{-}F)}$ = 20.2 Hz), 115.3, 62.1, 45.7, 14.5; HRMS (*m/z*): $[M+H]^+$ calcd. for $C_{20}H_{18}FN_6O_2$, 393.147; found, 393.1465.

2-Ethoxy-8-(5-fluoropyridin-3-yl)-9-[(4-{[(1*S*,4*S*)-5-methyl-2,5-diazabicyclo[2.2.1]heptan-2-yl]methyl}phenyl)methyl]-9*H*-purin-6-amine (Cpd 6: DSR-139293)

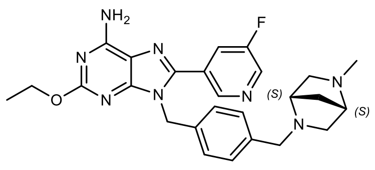

To a suspension of 4-{[6-amino-2-ethoxy-8-(5-fluoropyridin-3-yl)-9*H*-purin-9-yl]methyl}benzaldehyde (162 mg, 0.414 mmol) in THF (25 ml) were added (1*S*,4*S*)-2-methyl-2,5-diazabicyclo[2.2.1]heptane

dihydrobromide (171 mg, 0.622 mmol) and sodium acetate (103 mg, 1.26 mmol) at room temperature. After stirring at the same temperature for 30 min, sodium triacetoxyborohydride (175 mg, 0.826 mmol) added at room temperature. After stirring at the same temperature for 20 h, sodium triacetoxyborohydride (88.2 mg, 0.416 mmol) added at room temperature. After stirring at the same temperature for 2 h, the reaction mixture was quenched by satd. $NaHCO_3$aq. (20 ml) at 0 ºC and extracted with $CHCl_3$ (40 ml x2). The combined organic layer was dried over Na_2SO_4, filtered, and concentrated. The residue was purified by flash column chromatography (amino SiO_2, $CHCl_3$-MeOH; SiO_2, $CHCl_3$-MeOH, $CHCl_3$-MeOH-1-2% diethylamine) to give 2-ethoxy-8-(5-fluoropyridin-3-yl)-9-[(4-{[(1*S*,4*S*)-5-methyl-2,5-diazabicyclo[2.2.1]heptan-2-yl]methyl}phenyl)methyl]-9*H*-purin-6-amine (Cpd 6: DSR-139293) (129 mg, 64% yield) as white solids. m.p.: 155-157 °C; $[\alpha]^{D}_{23}$ = +16.6 (*c* = 0.97, $CHCl_3$); ^{1}H-NMR (400 MHz, $CDCl_3$): δ 8.64-8.62 p.p.m. (m, 2H), 8.51 (d, *J* = 2.8 Hz, 1H), 7.60-7.56 (m, 1H), 7.24 (d, *J* = 8.2 Hz, 2H), 6.99 (d, *J* = 8.2 Hz, 2H), 5.58 (brs, 2H), 5.38 (s, 2H), 4.38 (q, *J* = 7.2 Hz, 2H), 3.67 (d, *J* = 13.8 Hz, 1H), 3.60 (d, *J* = 13.8 Hz, 1H), 3.21-3.19 (m, 1H), 3.17-3.15 (m, 1H), 2.83 (d, *J* = 9.6, 1H), 2.67 (d, *J* = 10.1, 1H), 2.61 (dd, *J* = 2.3, 10.1 Hz), 2.56 (dd, *J* = 2.5, 9.9 Hz, 1H), 2.36 (s, 3H), 1.39 (t, *J* = 7.2 Hz, 3H); ^{13}C-NMR (100 MHz, $CDCl_3$): δ 162.4 p.p.m., 159.0 (d, $J_{(C\text{-}F)}$ = 259.1 Hz), 156.3, 154.0, 145.5 (d, $J_{(C\text{-}F)}$ = 1.9 Hz), 145.1 (d, $J_{(C\text{-}F)}$ = 3.9 Hz), 140.1, 139.1 (d, $J_{(C\text{-}F)}$ = 23.1 Hz), 134.4, 129.0 (2C), 127.7 (d, $J_{(C\text{-}F)}$ = 3.9 Hz), 126.6 (2C), 122.9 (d, $J_{(C\text{-}F)}$ = 19.3 Hz), 115.9, 63.7, 63.3, 62.0, 57.6, 57.6, 56.1, 46.7, 41.3, 33.4, 14.5; HRMS (*m/z*): $[M+H]^+$ calcd. for $C_{26}H_{30}FN_8O$, 489.2521; found, 489.2516; HPLC purity = 96.3% (Rt = 2.46 min).

Methyl 4-{[6-chloro-2-ethoxy-8-(5-fluoropyridin-3-yl)-9*H*-purin-9-yl]methyl}benzoate

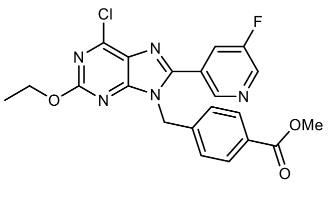

To a suspension of methyl 4-{[6-amino-2-ethoxy-8-(5-fluoropyridin-3-yl)-9*H*-purin-9-yl]methyl}benzoate (28.1 g, 66.5 mmol) and benzyltriethylammonium chloride (30.3 g, 133 mmol) in CH_2Cl_2 (450 ml) were

added slowly trimethylchlorosilane (84.4 ml, 665 mmol) and then *tert*-butyl nitrite (43.8 ml, 333 mmol) at reflux temperature. After stirring at reflux temperature for 4 h, the reaction mixture was added slowly over 20 min to a suspenstion of satd. $NaHCO_3$ aq. (1500 ml) at 0 ºC, stirred at the same temperature for 30 min, and extracted with $CHCl_3$ (1000 ml x2). The combined organic layer was dried over Na_2SO_4 and concentrated. The residue was suspended in MeOH (300 ml) and stirred at 50 ºC for 1 h. The suspension was stirred at the room temperature for 45 min and at 0 ºC for 1 h. The precipitates were filtered, washed with cooled MeOH (50 ml x 4), and dried in vacuo to afford methyl 4-{[6-chloro-2-ethoxy-8-(5-fluoropyridin-3-yl)-9*H*-purin-9-yl]methyl}benzoate (19.67 g, 44.5 mmol, 66.9% yield) as white solids. m.p.: 157-159 °C; ^{1}H-NMR (400 MHz, DMSO-d_6): δ 8.76-8.70 p.p.m. (m, 2H), 8.09-8.04 (m, 1H), 7.83 (d, J = 7.9 Hz, 2H), 7.20 (d, J = 7.9 Hz, 2H), 5.66 (s, 2H), 4.39 (q, J = 7.3 Hz, 2H), 3.80 (s, 3H), 1.33 (t, J = 7.3 Hz, 3H); ^{13}C-NMR (100 MHz, $CDCl_3$): δ 166.2 p.p.m., 161.2, 159.0 (d, $J_{(C\text{-}F)}$ = 260.1 Hz), 155.5, 152.2, 150.0 (d, $J_{(C\text{-}F)}$ = 1.9 Hz), 144.9 (d, $J_{(C\text{-}F)}$ = 3.9 Hz), 140.3 (d, $J_{(C\text{-}F)}$ = 23.1 Hz), 139.8, 130.5 (2C), 130.4, 127.1, 126.5 (2C), 126.4, 123.5 (d, $J_{(C\text{-}F)}$ = 20.2 Hz), 64.9, 52.3, 47.2, 14.3; HRMS (*m/z*): $[M+H]^+$ calcd. for $C_{21}H_{18}ClFN_5O_3$, 442.1077; found, 442.1075.

Methyl 4-{[2-ethoxy-8-(5-fluoropyridin-3-yl)-6-methyl-9*H*-purin-9-yl]methyl}benzoate

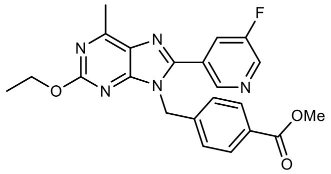

To a suspension of methyl 4-{[6-chloro-2-ethoxy-8-(5-fluoropyridin-3-yl)-9*H*-purin-9-yl]methyl}benzoate (19.67 g, 44.5 mmol) and bis(tri-*tert*-butylphosphine)palladium (1.137 g, 2.225 mmol) in THF (380 ml) was added methylzinc chloride (29 ml, 58.0 mmol) over 10 min at 4-10 ºC. After stirring at 4-6 ºC for 40 min, the reaction mixture was quenched by satd. NH_4Cl aq. (400 ml) at 0 ºC and extracted with $CHCl_3$ (500 ml x2). The combined organic layer was dried over Na_2SO_4 and concentrated. The residue was purified by flash column chromatography (SiO_2, $CHCl_3$-MeOH) to methyl 4-{[2-ethoxy-8-(5-fluoropyridin-3-yl)-6-methyl-9*H*-purin-9-yl]methyl}benzoate (18.0 g, 42.7 mmol, 96% yield) as pale yellow solids. m.p.: 123-

125 °C; ^{1}H-NMR (400 MHz, DMSO-d_6): δ 8.73-8.69 p.p.m. (m, 2H), 8.07-8.01 (m, 1H), 7.83 (d, J = 8.5 Hz, 2H), 7.15 (d, J = 8.5 Hz, 2H), 5.64 (s, 2H), 4.36 (q, J = 7.3 Hz, 2H), 3.80 (s, 3H), 2.71 (s, 3H), 1.31 (t, J = 7.3 Hz, 3H); ^{13}C-NMR (100 MHz, $CDCl_3$): δ 166.3 p.p.m., 161.9, 161.9, 159.0 (d, $J_{(C\text{-}F)}$ = 260.1 Hz), 154.4, 148.3 (d, $J_{(C\text{-}F)}$ = 1.9 Hz), 145.0 (d, $J_{(C\text{-}F)}$ = 3.9 Hz), 140.5, 139.8 (d, $J_{(C\text{-}F)}$ = 23.1 Hz), 130.4 (2C), 130.2, 128.7, 127.2 (d, $J_{(C\text{-}F)}$ = 3.9 Hz), 126.5 (2C), 123.3 (d, $J_{(C\text{-}F)}$ = 19.3 Hz), 63.8, 52.2, 46.7, 19.7, 14.5; HRMS (*m/z*): $[M+H]^+$ calcd. for $C_{22}H_{21}FN_5O_3$, 422.1623; found, 422.1616.

(4-{[2-Ethoxy-8-(5-fluoropyridin-3-yl)-6-methyl-9*H*-purin-9-yl]methyl}phenyl)methanol

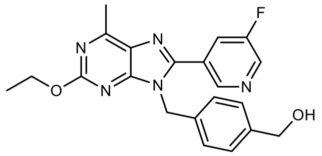

Synthetic procedure was followed as described for (4-{[6-amino-2-ethoxy-8-(5-fluoropyridin-3-yl)-9*H*-purin-9-yl]methyl}phenyl)methanol. The crude material was purified by flash column chromatography (SiO_2, $CHCl_3$-MeOH) to afford compound (4-{[2-ethoxy-8-(5-fluoropyridin-3-yl)-6-methyl-9*H*-purin-9-yl]methyl}phenyl)methanol (10.10 g, 25.7 mmol, 87% yield) as white solids. m.p.: 140-142 °C; ^{1}H-NMR (400 MHz, DMSO-d_6): δ 8.74-8.73 p.p.m. (m, 2H), 8.09-8.04 (m, 1H), 7.19 (d, J = 7.9 Hz, 2H), 6.95 (d, J = 7.9 Hz, 2H), 5.53 (s, 2H), 5.13 (t, J = 6.1 Hz, 1H), 4.39 (d, J = 6.1 Hz, 2H), 4.38 (q, J = 7.3 Hz, 2H), 2.70 (s, 3H), 1.33 (t, J = 7.3 Hz, 3H); ^{13}C-NMR (100 MHz, $CDCl_3$): δ 161.9 p.p.m., 161.8, 159.0 (d, $J_{(C\text{-}F)}$ = 259.1 Hz), 154.4, 148.4 (d, $J_{(C\text{-}F)}$ = 1.9 Hz), 145.2 (d, $J_{(C\text{-}F)}$ = 4.8 Hz), 141.1, 139.7 (d, $J_{(C\text{-}F)}$ = 22.2 Hz), 134.9, 128.7, 127.6 (2C), 127.4 (d, $J_{(C\text{-}F)}$ = 3.6 Hz), 126.8 (2C), 123.4 (d, $J_{(C\text{-}F)}$ = 19.3 Hz), 64.7, 63.8, 46.7, 19.7, 14.5; HRMS (*m/z*): $[M+H]^+$ calcd. for $C_{21}H_{21}FN_5O_2$, 394.1674; found, 394.1671.

4-{[2-Ethoxy-8-(5-fluoropyridin-3-yl)-6-methyl-9*H*-purin-9-yl]methyl}benzaldehyde

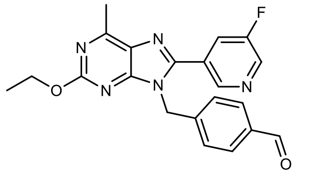

Synthetic procedure was followed as described for compound 4-{[6-amino-2-ethoxy-8-(5-fluoropyridin-3-yl)-9*H*-purin-9-yl]methyl}benzaldehyde. The crude material was purified by flash column chromatography (SiO_2, $CHCl_3$-MeOH) to afford compound 4-{[2-ethoxy-8-(5-fluoropyridin-3-yl)-6-methyl-9*H*-purin-9-yl]methyl}benzaldehyde (9.40 g, 24.02 mmol, 90% yield) as white solids. m.p.: 156-158 °C; ^{1}H-NMR (400 MHz, DMSO-d_6): δ 9.92 p.p.m. (s, 1H), 8.74-8.71 (m, 2H), 8.09-8.03 (m, 1H), 7.79 (d, J = 7.9 Hz, 2H), 7.23 (d, J = 7.9 Hz, 2H), 5.66 (s, 2H), 4.36 (q, J = 7.3 Hz, 2H), 2.71 (s, 3H), 1.31 (t, J = 7.3 Hz, 3H); ^{13}C-NMR (100 MHz, $CDCl_3$): δ 191.3 p.p.m., 162.0, 161.9, 159.0 (d, $J_{(C\text{-}F)}$ = 259.1 Hz), 154.4, 148.2 (d, $J_{(C\text{-}F)}$ = 1.9 Hz), 144.9 (d, $J_{(C\text{-}F)}$ = 3.9 Hz), 142.1, 139.9 (d, $J_{(C\text{-}F)}$ = 23.1 Hz), 136.2, 130.5 (2C), 128.7, 127.1 (2C), 127.1, 123.3 (d, $J_{(C\text{-}F)}$ = 19.3 Hz), 63.8, 46.7, 19.7, 14.5; HRMS (*m/z*): $[M+H]^+$ calcd. for $C_{21}H_{19}FN_5O_2$, 392.1517; found, 392.1513.

2-Ethoxy-8-(5-fluoropyridin-3-yl)-6-methyl-9-[(4-{[(1*S*,4*S*)-5-methyl-2,5-diazabicyclo[2.2.1]heptan-2-yl]methyl}phenyl)methyl]-9*H*-purine (Cpd 7: DSR-139970)

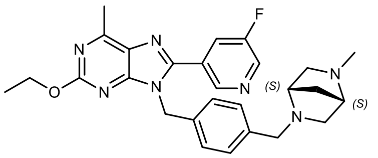

Synthetic procedure was followed as described for compound 2-ethoxy-8-(5-fluoropyridin-3-yl)-9-[(4-{[(1*S*,4*S*)-5-methyl-2,5-diazabicyclo[2.2.1]heptan-2-yl]methyl}phenyl)methyl]-9*H*-purin-6-amine (Cpd 6: DSR-139293). The crude material was purified by flash column chromatography (SiO_2, $CHCl_3$-MeOH) to afford compound 2-ethoxy-8-(5-fluoropyridin-3-yl)-6-methyl-9-[(4-{[(1*S*,4*S*)-5-methyl-2,5-diazabicyclo[2.2.1]heptan-2-yl]methyl}phenyl)methyl]-9*H*-purine (Cpd 7: DSR-139970) (10.20 g, 20.92 mmol, 70.6% yield) as a colorless gum. $[\alpha]^D{}_{25}$ = +16.0 (c = 1.05, $CHCl_3$); ^{1}H-NMR (400 MHz, DMSO-d_6): δ 8.73-8.71 p.p.m. (m, 2H), 8.05-8.01 (m, 1H), 7.18 (d, J = 7.9 Hz, 1H), 6.91 (d, J = 7.9 Hz, 2H), 5.52 (s, 2H), 4.38 (q, J = 7.3 Hz, 2H), 3.58 (d, J = 13.4 Hz, 1H), 3.51 (d, J = 13.4 Hz, 1H), 3.31 (s, 2H), 3.17 (s, 1H), 3.13 (s, 1H), 2.70 (s, 3H), 2.54-2.44 (m, 4H), 2.28 (s, 3H), 1.59 (s, 2H), 1.33 (t, J = 7.3 Hz, 3H); ^{13}C-NMR (100 MHz, $CDCl_3$): δ 161.8 p.p.m., 161.7, 159.0 (d, $J_{(C\text{-}F)}$ = 259.1 Hz), 154.4, 148.5 (d, $J_{(C\text{-}F)}$ = 1.9

Hz), 145.3 (d, $J_{(C-F)}$ = 3.9 Hz), 140.3, 139.6 (d, $J_{(C-F)}$ = 23.1 Hz), 134.1, 129.1 (2C), 128.7, 127.5 (d, $J_{(C-F)}$ = 3.9 Hz), 126.6 (2C), 123.4 (d, $J_{(C-F)}$ = 20.2 Hz), 63.8, 63.7, 62.0, 57.6 (2C), 56.2, 46.8, 41.3, 33.4, 19.7, 14.5; HRMS (*m/z*): $[M+H]^+$ calcd. for $C_{27}H_{31}FN_7O$, 488.2569; found, 488.2564; HPLC purity = 97.8% (Rt = 2.70 min).

7-(3-methylphenyl)pyrazolo[1,5-*a*]pyrimidine-3-carboxamide (CU-CPT8m)

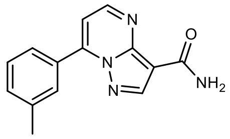

CU-CPT8m was prepared according to the literature as white solids [6]. m.p.: 224-226 °C; ^{1}H-NMR (400 MHz, $CDCl_3$): δ 8.71 p.p.m. (s, 1H), 8.64 (d, *J* = 4.3 Hz, 1H), 7.99 (brs, 1H), 7.82-7.78 (m, 2H), 7.49-7.44 (m, 1H), 7.42-7.38 (m, 1H), 7.04 (d, *J* = 4.9 Hz, 1H), 5.62 (brs, 1H), 2.46 (s, 3H); ^{13}C-NMR (100 MHz, DMSO-d_6): δ 162.6 p.p.m., 151.8, 147.3, 146.8, 145.7, 137.9, 132.1, 130.0, 129.8, 128.4, 126.9, 109.0, 105.3, 21.0; HRMS (*m/z*): $[M+H]^+$ calcd. for $C_{14}H_{13}N_4O$, 253.1084; found, 253.1084; HPLC purity = 96.4% (Rt = 3.56 min).

Reference

1. K. Hirota *et al.*, Discovery of 8-hydroxyadenines as a novel type of interferon inducer. *Journal of medicinal chemistry* **45**, 5419 (2002).

2. A. Kurimoto *et al.*, Synthesis and evaluation of 2-substituted 8-hydroxyadenines as potent interferon inducers with improved oral bioavailabilities. *Bioorganic & medicinal chemistry* **12**, 1091 (2004)

3. Y. Isobe *et al.*, Preparation of adenine derivatives as interferon inducing agents, antiviral agents, anticancer agents, etc.. WO2008114817.

4. K. Hirota *et al.*, Efficient synthesis of 2,9-disubstituted 8-hydroxyadenine derivatives. *Organic & biomolecular chemistry* **1**, 1354 (2003).

5. Y. Isobe and T. Nakamura, Preparation of adenine compounds as Toll-like receptor (TLR) agonists.

WO2008114817.

6. S. Zhang *et al.*, Small-molecule inhibition of TLR8 through stabilization of its resting state. *Nature chemical biology* **14**, 58-64. (2018).
